# Inter‐ and intra‐tumoural heterogeneity in cancer‐associated fibroblasts of human pancreatic ductal adenocarcinoma

**DOI:** 10.1002/path.5224

**Published:** 2019-02-22

**Authors:** Cindy Neuzillet, Annemilaï Tijeras‐Raballand, Chanthirika Ragulan, Jérôme Cros, Yatish Patil, Matthieu Martinet, Mert Erkan, Jörg Kleeff, Jeremy Wilson, Minoti Apte, Marie Tosolini, Abigail S Wilson, Francesca R Delvecchio, Corinne Bousquet, Valérie Paradis, Pascal Hammel, Anguraj Sadanandam, Hemant M Kocher

**Affiliations:** ^1^ Centre for Tumour Biology, Barts Cancer Institute ‐ a CRUK Centre of Excellence Queen Mary University of London London UK; ^2^ Barts and The London HPB Centre The Royal London Hospital Barts Health NHS Trust, London UK; ^3^ INSERM UMR1149 Beaujon University Hospital, Paris 7 Diderot University Paris France; ^4^ Department of Medical Oncology Curie Institute, Versailles Saint‐Quentin University Paris France; ^5^ AFR Oncology Paris France; ^6^ Division of Molecular Pathology The Institute of Cancer Research London UK; ^7^ Centre for Molecular Pathology The Royal Marsden Hospital NHS Foundation Trust London UK; ^8^ Department of Pathology Beaujon University Hospital, Paris 7 Diderot University Paris France; ^9^ Department of Surgery Koc University School of Medicine Istanbul Turkey; ^10^ Department of Visceral, Vascular and Endocrine Surgery Martin‐Luther‐University Halle‐Wittenberg Halle (Saale) Germany; ^11^ Pancreatic Research Group, South Western Sydney Clinical School University of New South Wales and Ingham Institute for Applied Medical Research Sydney Australia; ^12^ INSERM UMR 1037, Technological Pole and Bioinformatic Platform Cancer Research Center of Toulouse Toulouse France; ^13^ INSERM UMR 1037, Team 6 Protein Synthesis and Secretion in Carcinogenesis Cancer Research Center of Toulouse Toulouse France; ^14^ Digestive Oncology Unit Beaujon University Hospital, Paris 7 Diderot University Paris France

**Keywords:** pancreatic stellate cell, stroma, transcriptomics, tumour microenvironment, tumour‐stroma interactions

## Abstract

Cancer‐associated fibroblasts (CAF) are orchestrators of the pancreatic ductal adenocarcinoma (PDAC) microenvironment. Stromal heterogeneity may explain differential pathophysiological roles of the stroma (pro‐ versus anti‐tumoural) in PDAC. We hypothesised that multiple CAF functional subtypes exist in PDAC, that contribute to stromal heterogeneity through interactions with cancer cells. Using molecular and functional analysis of patient‐derived CAF primary cultures, we demonstrated that human PDAC‐derived CAFs display a high level of inter‐ and intra‐tumour heterogeneity. We identified at least four subtypes of CAFs based on transcriptomic analysis, and propose a classification for human PDAC‐derived CAFs (pCAFassigner). Multiple CAF subtypes co‐existed in individual patient samples. The presence of these CAF subtypes in bulk tumours was confirmed using publicly available gene expression profiles, and immunostainings of CAF subtype markers. Each subtype displayed specific phenotypic features (matrix‐ and immune‐related signatures, vimentin and α‐smooth muscle actin expression, proliferation rate), and was associated with an assessable prognostic impact. A prolonged exposure of non‐tumoural pancreatic stellate cells to conditioned media from cancer cell lines (cancer education experiment) induced a CAF‐like phenotype, including loss of capacity to revert to quiescence and an increase in the expression of genes related to CAF subtypes B and C. This classification demonstrates molecular and functional inter‐ and intra‐tumoural heterogeneity of CAFs in human PDAC. Our subtypes overlap with those identified from single‐cell analyses in other cancers, and pave the way for the development of therapies targeting specific CAF subpopulations in PDAC. © 2018 The Authors. *The Journal of Pathology* published by John Wiley & Sons Ltd on behalf of Pathological Society of Great Britain and Ireland.

## Introduction

Pancreatic ductal adenocarcinoma (PDAC) is characterised by an abundant desmoplastic stroma, a complex structure composed of ECM proteins and various cell types including cancer‐associated fibroblasts (CAF), immune cells, and endothelial cells 1. CAFs are orchestrators of the PDAC microenvironment: they are responsible for excess ECM production and interact with both cancer cells and other stromal cells through a network of signalling pathways and mediators 2, 3. These interactions promote tumour growth, invasion, metastasis, and resistance to therapy 1, 2. A major source of CAFs in PDAC are pancreatic stellate cells (PSC), which are resident mesenchymal cells of the pancreas that, in their quiescent state, store vitamin A‐containing lipid droplets 4, 5. Upon activation, PSCs lose this storage function, express α‐smooth muscle actin (αSMA), and secrete ECM proteins and pro‐tumoural factors 2, 3, 5. The dynamics between non‐tumoural PSCs and CAFs and their plasticity remain scarcely explored 6, 7.

Recently, the role of the stroma and CAFs in PDAC has been questioned, by indications that CAFs may restrain rather than promote tumour growth 8, 9. Non‐selective genetic disruption of CAFs, using αSMA‐positive cell depletion 8 or pharmacological inhibition of the sonic hedgehog pathway 9, 10, yielded aggressive tumours in mice and clinical trial failures, suggesting that some CAF subpopulations may be protective, and highlighting that caution should be exercised when targeting the stroma in PDAC.

Inter‐tumoural molecular heterogeneity of cancer cells in PDAC is well‐established 11, 12, 13, 14. Phenotypic stromal features (i.e. abundance of fibrosis and immune cell infiltration) also vary across tumours 15. Because CAFs are at the crossroads of stromal compartments in PDAC, we hypothesised that the inter‐tumour stromal heterogeneity may be related to patient‐specific profiles of CAFs. Although there is increasing evidence for CAF heterogeneity in various cancers 6, 16, data about functional heterogeneity of CAFs in PDAC remain limited to murine experiments, mainly due to experimental challenges (i.e. difficulty of expanding primary cultures, small quantity of material, lack of subtype markers for cell sorting in PDAC) 7, 17.

## Materials and methods

### Patient consent and ethical approval

Ethics approval was obtained for the use of patient tumour samples. German contribution (human primary cultures): Ethics Committee of the Faculty of Medicine of the Technical University of Munich, number 1926/07; first approved 30 October 2007. Australian contribution (human primary cultures): institutional ethics approval number HREC11189/SESIAHS 00/088. French contribution (FFPE tumour samples): Beaujon biobank registration number BB‐0033‐00078. UK contribution (pancreatic stellate cells): UK Human Tissue Bank; Trent MREC, 05/MRE04/82. All participants gave informed consent before taking part.

### Primary CAFs

Primary CAF cultures were isolated using the previously described outgrowth method (M. Apte's and M. Erkan's groups) 18. All experiments for functional and molecular characterisation of CAFs were performed on a single passage for each CAF culture. All care was taken to minimise the effect of cell growth in artificial conditions by using early passages.

### RNA analysis

The PanCancer Progression panel of genes was profiled using the nCounter^®^ Max Analysis System (NanoString Technologies, Seattle, CA, USA). Data quality and normalisation were performed using nSolver analysis software (NanoString Technologies) as per the manufacturer's instructions and as described by us 19.

### Subtype and signature identification

Gene expression profiles were clustered using a consensus non‐negative matrix factorisation (NMF) approach using the R package NMF 20. Prediction analysis of microarrays (PAM) was used to assign genes to specific subtypes using centroids, as described previously 21.

### Patient dataset and pCAFassigner subtypes

The International Cancer Genome Consortium (ICGC) dataset from Bailey *et al* 12, comprising 96 pancreatic samples (RNAseq), was used to assign pCAFassigner subtypes using PAM centroids generated from CAF primary cultures. The subtypes were assigned to the patient samples by correlating the PAM centroids and their corresponding gene expression (Pearson correlation) for each sample from the ICGC dataset. The subtypes were assigned based on highest correlation coefficient 22. Potential second and third subtypes were assigned where the correlation coefficients were second and third highest and not negative. After subtype assignment, only PDAC samples (*n* = 70) were selected for the patient survival analysis.

### Cancer‐education experiment

MIAPaCa‐2 and AsPC‐1 cells were plated and split every 3 days at a fixed cell number. Before splitting, conditioned medium (CM) was taken and filtered using a 0.22‐μm filter. In parallel, three flasks with a fixed number of PS1 cells were plated and split every 6 days: (1) in usual PS1 medium; (2) in a 1:1 mix of fresh PS1 medium and MIAPaCa‐2‐CM; and (3) in a 1:1 mix of fresh PS1 medium and AsPC‐1‐CM, in order to obtain concurrent relevant educated PSCs with parental (control) PSCs with similar time lapsed in tissue‐culture. Freshly harvested cancer cell CM was used to avoid alterations induced by freeze/thawing cycles. The same batch of FBS (Sigma‐Aldrich, St. Louis, MO, USA) was used over the whole experiment. After 2 months of culture, parental and educated PS1 cells were harvested for analyses. Cells were then cultured in standard PS1 medium for 1 month to test for reversibility.

### Statistical analyses

Experiments were performed in triplicates with immortalised cell lines. For primary cells, we obtained at least two primary cultures for each subtype, and analysed them to describe the biological properties of each pCAFassigner subtype. Unless otherwise stated, unpaired Student *t*‐tests with Welch's correction were used to compare two groups for continuous variables. Non‐parametric one‐way ANOVA using Kruskal–Wallis tests and Dunn's multiple comparisons tests were performed to compare more than two groups. Results are expressed as mean ± SD. Survival curves were estimated with the Kaplan–Meier method and compared using the log‐rank test. The level of significance for all tests was *p* < 0.05. Data were analysed using Prism software v.6.0 (GraphPad Software Inc, San Diego, CA, USA).

More details are provided in supplementary material, Supplementary materials and methods.

## Results

### Transcriptomic analysis reveals inter‐tumour heterogeneity of CAF primary cultures

To test the hypothesis that PDAC‐derived CAFs display inter‐tumoural heterogeneity, we grew 16 primary CAF cultures from 16 different PDAC patients in the UK, Germany, and Australia (see supplementary material, Table S1) and profiled them for 770 genes using the Nanostring nCounter Cancer Progression panel, which was appropriate for stromal gene expression, including ECM and epithelial‐to‐mesenchymal transition genes. The absence of contamination by cancer cells in the CAF cultures was verified by the lack of ubiquitous tumoural *KRAS* mutations (see supplementary material, Figure S1A, Table S2).

**Figure 1:**
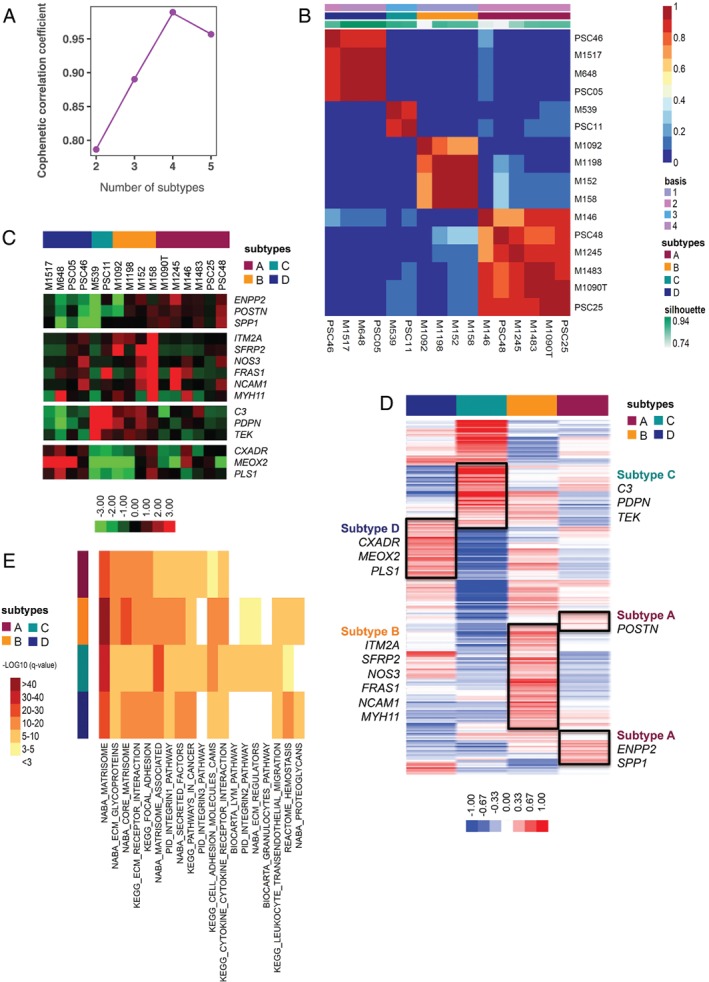
PDAC CAF classification (pCAFassigner). (A) Cophenetic correlation plot for *k* = 2 to *k* = 5 classes after NMF for transcriptome of the 16 patient‐derived CAF primary cultures. The maximum cophenetic coefficient value was reached for *k* = 4 classes (>0.99). (B) Consensus matrix clustering after NMF for transcriptome of the 16 patient‐derived CAF primary cultures for *k* = 4 classes. (C) Heatmap with hierarchical clustering for 15 selected metagenes that were found most discriminating between patient‐derived CAF primary cultures (short pCAFassigner). Significantly higher expression is shown in red and lower expression in green. (D) Heatmap showing 248 metagenes (extended pCAFassigner) between CAF subtypes, based on PAM‐derived centroids. Significantly higher expression is shown in red and lower expression in blue. (E) Gene expression pathways using mSigDB database 54. We selected genes from the extended pCAFassigner with a PAM centroid value >0.10 in each CAF subtype. Top‐10 pathways for each CAF subtype are displayed. Significantly lower *q*‐value (i.e. FDR‐adjusted *P* value) is shown in red and higher *q*‐value in yellow/white.

Initial unsupervised NMF clustering of highly variable 248 genes (SD > 0.8 across samples) from these CAF cultures defined four optimal CAF subtypes (pCAFassigner [pCAF] subtypes A–D; cophenetic coefficient > 0.99; Figure 1A,B; see supplementary material, Figure S1B–D). The robustness of the four‐cluster model was further validated using silhouette width and consensus clustering of samples after variable gene selection approach (see supplementary material, Figures S1D,E, S2A–C). The four subtypes were characterised by distinct mRNA expression profiles (see supplementary material, Figure S2D) with the 15 most discriminating genes used for further validation (Figure 1C). Supervised clustering analysis using PAM‐derived centroids (summary of gene expression per subtype) assigned the expression of the 248 genes to specific pCAF subtypes (Figure 1D). These results suggest that, amongst these primary human PDAC CAF cultures, at least four subtypes exist.

Pathway enrichment analyses revealed that pCAF subtypes displayed partially overlapping pathways, with a significant enrichment (*q*‐value, i.e. false discovery rate [FDR]‐adjusted *P* value) of ECM‐related gene sets across all subtypes, while subtype C expressed immune‐related pathways that were not found in other subtypes (Figure 1E). This finding suggested that pCAF subtypes are functionally distinct.

### Multiple CAF subtypes co‐exist within each tumour sample

**Figure 2:**
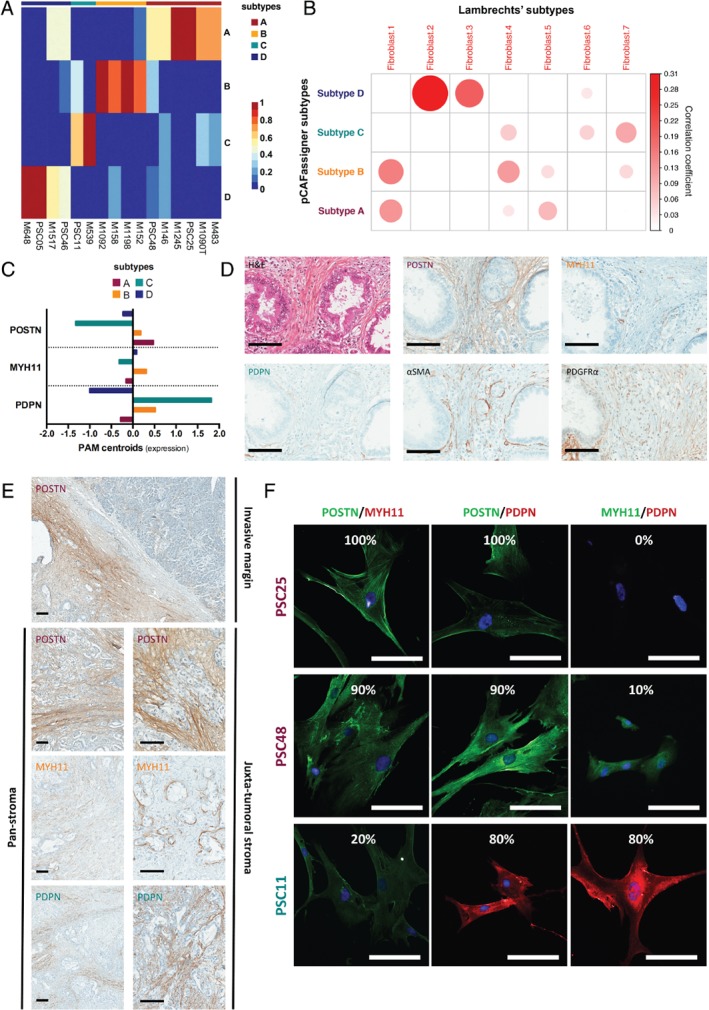
Molecular markers for PDAC CAF. (A) Heatmap of CAF culture (*n* = 16) probability of belonging to pCAFassigner subtypes. Significantly higher probability is shown in red and lower probability in blue. (B) Correlation of pCAFassigner subtype PAM centroids with Lambrechts *et al* 16 fibroblast subtype gene expression. Only positive correlations were shown. (C) PAM centroids (expression levels) of *POSTN*, *MYH11*, and *PDPN* according to pCAFassigner subtypes. (D) H&E stain and immunohistochemical staining for periostin (POSTN), myosin‐11 (MYH11), podoplanin (PDPN), αSMA and PDGFRα on serial sections from a resected PDAC sample. Scale bars: 100 μm. (E) Representative pictures of IHC staining for periostin (POSTN), myosin‐11 (MYH11) and podoplanin (PDPN) in human PDAC samples, showing spatial pattern at the invasive margin and in the juxta‐tumoural stroma and pan‐stroma. Scale bar: 100 μm. (F) Immunofluorescence co‐staining of POSTN (green), MYH11 (red or green), PDPN (red) and DAPI (blue) on PSC25 (subtype A), PSC48 (mixed, subtype A dominant > B) and PSC11 (subtype C) (merged images). Percentages of positive cells for each marker are displayed. Scale bar: 100 μm.

Each CAF culture was assigned to one particular pCAF subtype based on the consensus clustering approach and predominant population according to the NMF's highest probability score (Figure 2A). Our subtype clustering profile supported the hypothesis of multiple subpopulations (i.e. intra‐tumour heterogeneity) within single patient‐derived CAF cultures. Recently, Lambrechts *et al* 16 described seven clusters of fibroblasts in normal lung and cancer microenvironment, using a single‐cell RNAseq approach. We could demonstrate that PAM centroids from our pCAFassigner classification correlated with the Lambrechts classification (Figure 2B). Indeed, we observed that pCAFassigner subtypes showed overlap with multiple Lambrechts subtypes, supporting the notion of intra‐tumoural heterogeneity. pCAF subtype A was primarily correlated with fibroblast 1 and 5 subtypes of Lambrechts *et al*, subtype B with fibroblast 1 and 4, subtype C with fibroblast 7 and subtype D with fibroblast 2 and 3. In addition, pCAFassigner subtype‐specific genes showed specific clustering in Lambrechts *et al* subtypes (see supplementary material, Figure S3A).

We next sought to explore the CAF intra‐tumour heterogeneity within human PDAC samples. We screened antibodies for detection by IHC of the top 15 markers (Figure 1C) previously identified from the pCAFassigner. We selected markers that fulfilled the following criteria: (1) high mRNA expression in a specific subtype and low or no expression in other subtypes (Figures 1C and 2C, see supplementary material, Figure S2D), (2) strong or intermediate expression in fibroblasts by IHC, (3) no or low expression by other stromal cells, unless part of an easily identifiable anatomical structure (e.g. artery, nerve), and (4) no or low expression by cancer cells. POSTN (periostin), MYH11 (myosin‐11), and PDPN (podoplanin) were selected as pCAF subtype A‐, B‐, and C‐related markers, respectively (Figure 2C–E, supplementary material, Figure S3B). No marker fulfilled all these criteria for pCAF subtype D (*CXADR* and *MEOX* were strongly expressed by tumour cells; *PLS1* by endothelial cells).

Immunohistochemistry (IHC) on serial sections from patient surgical samples showed the presence of POSTN, PDPN and MYH11 in spatially distinct areas of the tumour, suggesting expression of these markers by different CAF subpopulations (Figure 2D,E and supplementary material, Figure S3B). POSTN was present both at the invasive margin and in the centre of the tumours (juxta‐tumoural stroma [<100 μm] and pan‐stroma 23), as previously reported 24, while MYH11 and PDPN were found only in the centre (juxta‐tumoural stroma and pan‐stroma) (Figure 2E). In addition, we identified platelet‐derived growth factor receptor α (PGDFRα) as a potential pan‐subtype CAF marker, as described previously 25, whereas αSMA expression was not found in all CAFs 7 and was not specific to one particular subtype population (Figure 2D and supplementary material, Figure S3B).

To rule out the hypothesis of co‐expression of several markers by single cells, we performed an additional co‐immunofluorescence experiment for POSTN, MYH11 and PDPN on three primary CAF cultures: PSC25 (subtype A), PSC48 (mixed, subtype A dominant > B) and PSC11 (subtype C) (Figure 2F and supplementary material, Figure S3C). In these primary cultures, there was predominant staining of the marker associated with the pCAFassigner subtype (POSTN in PSC25 and PSC48, PDPN in PSC11) over other markers. Furthermore, the other pCAFassigner markers were expressed focally in distinct cells (i.e. single marker expression, no overlap) at a much lower intensity or no expression at all. These findings were consistent with our transcriptomic results. Overall, these *in vitro* co‐staining data again supported the co‐existence of *in vivo* spatially distinct CAF populations within single tumours.

The preponderance of pCAF subtype A amongst CAF primary cultures (*n* = 6/16, 37.5%; Figure 2A) may be due to predominance of this subtype within patient samples (Figure 2D,E and supplementary material, Figure S3B) or alternatively a technical artefact (i.e. the outgrowth method preferentially favours subtype A). To explore this hypothesis, we validated externally the presence of the CAFs subtypes at the mRNA level (by applying our pCAFassigner signatures and PAM centroids to bulk RNAseq gene expression data available from the ICGC 12, *n* = 70) and at the protein level (by IHC in Beaujon cohort, *n* = 50) *in situ* in PDAC samples. Consistent with primary cultures, subtype A CAFs were the most frequently represented subtype in the ICGC dataset (proportion of samples with subtype A as first or second subtypes: 40%) and in the IHC cohort (proportion of samples with high POSTN [subtype A marker] expression: 54%), supporting both inter‐ and intra‐tumoural heterogeneity of CAFs and subtype A predominance, and, moreover, confirming that *ex vivo* culture had a limited impact (see supplementary material, Tables S3–S5).

### CAF subtypes have a prognostic impact

**Figure 3:**
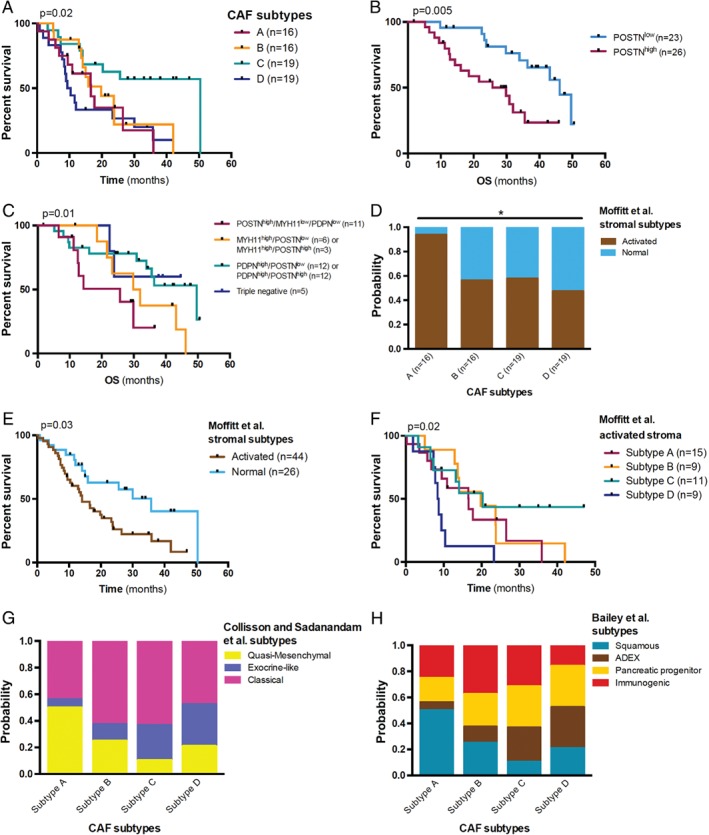
Prognostic impact of PDAC CAF subtypes. (A) Kaplan–Meier curves for OS in the ICGC dataset (*n* = 70 PDAC samples with RNAseq data from bulk tumour tissue). Subtype A is displayed in red, B in orange, C in green, and D in blue. Log‐rank tests, overall: *p* = 0.02; subtype C versus others: *p* = 0.004; subtype D versus others: *p* = 0.03; other comparisons: N.S. (B) Kaplan–Meier curves for OS according to periostin (POSTN) expression level by IHC (*n* = 49). High POSTN expression was defined as moderate or strong staining in >50% of stromal surface. Median OS: 29.8 months versus 46.2 months, in POSTN^high^ versus POSTN^low^ group, respectively. Log‐rank test, *p* = 0.005. (C) Kaplan–Meier curves for OS according to combined POSTN, myosin‐11 (MYH11) and podoplanin (PDPN) expression level by IHC (*n* = 49). High POSTN expression was defined as moderate or strong staining in >50% of stromal surface. High MYH11 and PDPN expressions were defined as the presence of strong stromal staining. In case of simultaneous high expression of MYH11 and PDPN, the tumour was classified according to the most abundant subpopulation. Median OS in POSTN^high^/MYH11^low^/PDPN^low^ (red): 25.7 months, MYH11^high^/POSTN^low^ or MYH11^high^/POSTN^high^ (orange): 30.9 months, PDPN^high^/POSTN^low^ or PDPN^high^/POSTN^high^ (green): 49.6 months, triple negative (POSTN^low^/MYH11^low^/PDPN^low^, blue): undefined. Log‐rank test for trend, *p* = 0.01. (D) Association between pCAFassigner subtypes and Moffitt *et al* stroma subtypes in the ICGC dataset (*n* = 70) as assessed by NTP analysis. Chi‐square test, *p* = 0.03. (E) Kaplan–Meier curves for OS according to Moffitt *et al* stroma subtypes in the ICGC dataset (*n* = 70). Median OS: 14.1 months versus 35.8 months in activated versus normal stroma, respectively. Log‐rank test, *p* = 0.03. (F) Kaplan–Meier curves for OS according to CAF subtypes in the Moffitt *et al* activated stroma group from the ICGC dataset (*n* = 44). Log‐rank test, *p* = 0.02. Median OS in subtype A: 16.6 months, subtype B: 19.8 months, subtype C: 20.3 months, and subtype D: 8.6 months. (G) Association between pCAFassigner subtypes and Collisson *et al* 11 tumour subtypes in the ICGC dataset (*n* = 70) as assessed by NTP analysis. Chi‐square test, *p* = 0.12 (overall), *p* = 0.03 (subtype A versus others). (H) Association between pCAFassigner subtypes and Bailey *et al* 12 tumour subtypes in the ICGC dataset (*n* = 70) as assessed by NTP analysis. Chi‐square test, *p* = 0.24 (overall), *p* = 0.06 (subtype A versus others).

We next considered the dominant pCAFassigner subtype in each patient sample within the ICGC cohort to explore the impact on survival (Figure 3A). We observed a significant difference in overall survival (OS) between the four pCAFassigner subtypes (*p* = 0.02) (Figure 3A). Subtype D‐dominant samples had the poorest prognosis (*p* = 0.03), with a median OS of 9.9 months, while patients with dominant subtype C had prolonged survival (*p* = 0.004), with a median OS of 50.4 months, and those with dominant subtype A or B had a poor/intermediate prognosis (median OS of 16.6 and 19.8 months, respectively) (Figure 3A and supplementary material, Table S3).

We then assessed the prognostic impact of POSTN, MYH11 and PDPN levels by IHC in the Beaujon cohort. High POSTN expression (defined as moderate or strong staining in >50% of stromal surface) was associated with significantly shorter OS (median: 29.8 versus 46.2 months, *p* = 0.005) (Figure 3B). In addition, combined POSTN, MYH11, and PDPN status defined three risk groups with poor (POSTN only/subtype A‐like, median OS: 25.7 months), intermediate (MYH11 ± POSTN/subtype B‐like, median OS: 30.9 months), and good (PDPN ± POSTN/subtype C‐like, median OS: 49.6 months) prognosis (*p* = 0.01) (Figure 3C).

Furthermore, we categorised our pCAFassigner subtypes from the ICGC data into Moffitt *et al* activated versus normal stromal subtypes 13 using their signature and nearest template prediction (NTP) statistical analysis. We observed that subtype A samples were enriched for ‘activated stroma’ signature (>90% versus ≈50% in other subtypes, *p* = 0.03) (Figure 3D), a signature associated with shorter survival (*p* = 0.030) (Figure 3E). Interestingly, we identified a favourable profile of subtype C over other subtypes within ‘activated stroma’ tumours (*p* = 0.02) (Figure 3F) and the ‘normal stroma’ group (see supplementary material, Figure S3D).

We next sought to explore the associations with known transcriptomic tumour subtypes (those of Collisson *et al* 11 and Bailey *et al* 12, Figure 3G–H). Similarly to Moffitt/pCAFassigner subtype association, poor prognostic (QM‐PDA/squamous) tumour subtypes were more frequently observed in samples with dominant subtype A CAFs (50% versus 18.5%, *p* = 0.03 and *p* = 0.06, respectively), suggesting a specific tumour‐stromal interaction associated with adverse outcome.

### CAF subtypes display specific molecular and functional features

Since pCAF subtype A displayed specific spatial expression pattern (Figure 2D) and prognostic features (Figure 3A–D,G, H), we next compared the primary CAF cultures belonging to subtype A versus other subtypes for molecular and functional features, using the well‐characterised immortalised stellate cell line (PS1) derived from normal pancreas and MRC5 human embryonic lung fibroblasts as standard references 26.

**Figure 4:**
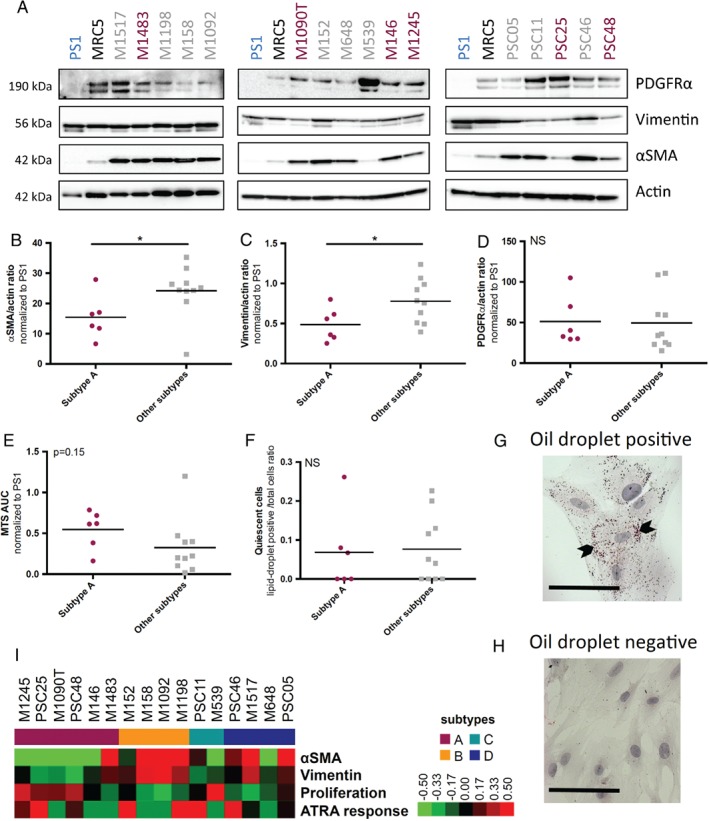
Phenotypic features of PDAC CAF subtypes. (A) αSMA, vimentin, PDGFRα, and β‐actin (actin) expression in CAF primary cultures (*n* = 16) and PS1 and MRC5 (human embryonic lung fibroblast) cell lines (used as controls) by western blot. Subtype A CAFs are displayed in red and other subtypes in grey. (B) Quantification of αSMA expression normalised to β‐actin (actin) using ImageJ (National Institute of Health, (Bethesda, MA, USA), according to pCAFassigner subtype (*n* = 16). αSMA mean expression normalised to PS1: 15.4 ± 7.2 in subtype A versus 24.2 ± 8.5 in other subtypes, unpaired *t*‐test with Welch's correction, *p* = 0.048. (C) Quantification of vimentin expression normalised to β‐actin (actin) using ImageJ, according to pCAFassigner subtype (*n* = 16). Vimentin mean expression normalised to PS1: 0.49 ± 0.21 in subtype A versus 0.78 ± 0.27 in other subtypes, unpaired *t*‐test with Welch's correction, *p* = 0.031. (D) Quantification of PDGFRα expression normalised to β‐actin (actin) using ImageJ, according to pCAFassigner subtype (*n* = 16). PDGFRα mean expression normalised to PS1: 51.2 ± 30.4 in subtype A versus 49.6 ± 35.0 in other subtypes, unpaired *t*‐test with Welch's correction, *p* = 0.92. (E) AUC assessed by MTS assay in CAF primary cultures, according to pCAFassigner subtype (*n* = 16). Mean AUC normalised to PS1: 0.55 ± 0.23 in subtype A versus 0.32 ± 0.34 in other subtypes, unpaired *t*‐test with Welch's correction, *p* = 0.15. (F) Ratio of lipid‐droplet‐positive (quiescent) cells over total cells, assessed by Oil Red O staining, in all‐trans retinoic acid (ATRA)‐treated CAF primary cultures (1 μm daily, for 5–7 days, until confluency; *n* = 16). Mean ratio: 0.068 ± 0.10 in subtype A versus 0.076 ± 0.087 in other subtypes, unpaired *t*‐test with Welch's correction, *p* = 0.87. (G,H) Representative images of ATRA‐responsive (positive) cells (G) and ATRA‐non‐responsive (negative) cells (H) after Oil Red O staining. (I) Heatmap summarising primary CAF culture (*n* = 16) features in terms of αSMA (αSMA/actin ratio by western blot), vimentin (vimentin/actin ratio by western blot) expression, proliferation (AUC of MTS curve), and ATRA response (lipid‐droplet‐positive cells/total cells ratio). All values were normalised to PS1 as a reference. Significantly higher values are shown in red and lower values in green.

Subtype A CAF cultures displayed low expression of the activation marker αSMA (*p* = 0.048) and low vimentin expression (*p* = 0.031) by western blot, compared to other pCAF subtypes (Figure 4A–C and supplementary material, Figure S4A,B). αSMA and vimentin high/low status were also validated by immunofluorescence (see supplementary material, Figure S4D,E). In contrast, PDGFRα was not differentially expressed between these two CAF groups (*p* = 0.92), which confirmed its status as a pan‐CAF marker (Figure 4A,D and supplementary material, Figure S4C) 27. In addition, subtype A pCAF cultures displayed a trend for higher proliferative activity assessed by MTS assay (*p* = 0.15) (Figure 4E and supplementary material, Figure 4F,G). In contrast, no difference was observed in terms of reversion to quiescence upon all‐trans retinoic acid (ATRA) treatment between the two groups (*p* = 0.87) (Figure 4F–H and supplementary material, Figure S4H). Similarly, no difference was observed in terms of cell size, both subtype A and other subtype CAF culture cells being significantly larger than non‐tumoural stellate cells (*p* < 0.001) (see supplementary material, Figure S4I). These phenotypic features are summarised in Figure 4I.

### CAF subtypes differentially affect cancer cells

**Figure 5:**
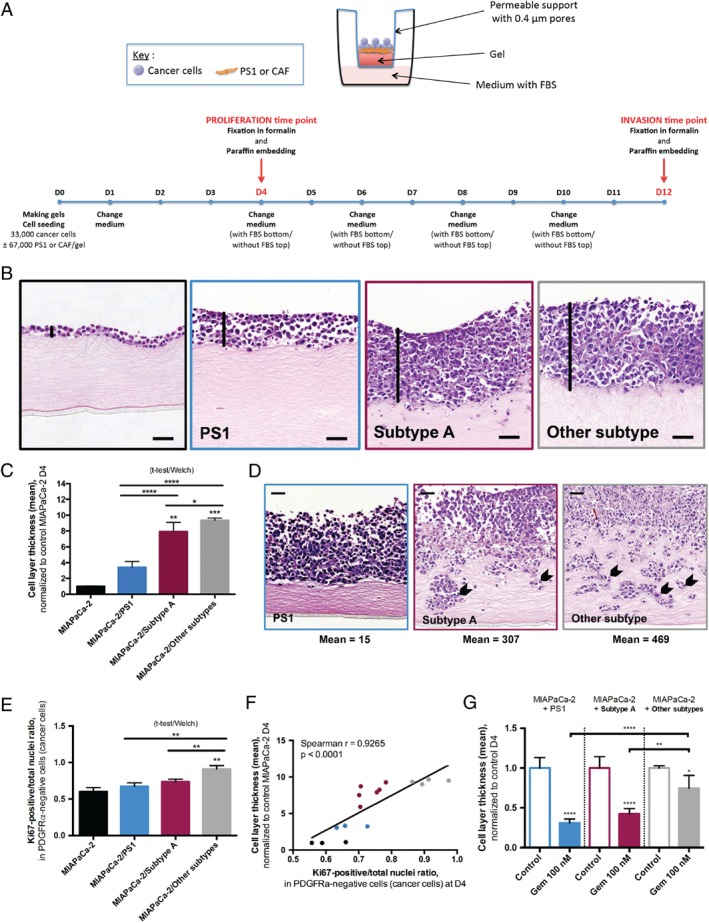
Influence of cancer‐associated fibroblast (CAF) subtypes on cancer cells. (A) Overview of the mini‐organotypic experimental system and timelines. (See supplementary material, Supplementary Methods, for detailed description.) (B) Representative images of H&E‐stained sections of mini‐organotypics after 4 days (D4), with MIAPaCa‐2 cells alone (top left), or in co‐culture with PS1 (top right), subtype‐A CAFs (bottom left) or other‐subtype CAFs (bottom right). Black vertical lines highlight the cell layer thickness. Scale bar: 50 μm. (C) Cell proliferation at D4 assessed by the cell layer thickness, measured at two representative points per field in an average of 3 fields with 10 × magnification on H&E‐stained slides (one mean value per gel). Mean cell layer thickness normalised to MIAPaCa‐2 alone: 1.00 ± 0.06 in MIAPaCa‐2 alone (triplicate), 3.41 ± 0.74 in MIAPaCa‐2/PS1 co‐culture (triplicate), 7.93 ± 1.16 in MIAPaCa‐2/subtype A CAF co‐culture (*n* = 2 distinct CAF cultures) and 9.39 ± 0.27 in MIAPaCa‐2/other‐subtype CAF co‐culture (*n* = 2 distinct CAF cultures), Kruskal–Wallis: *p* < 0.0001. Dunn's multiple comparisons: MIAPaCa‐2 alone versus MIAPaCa‐2/subtype A: *p* < 0.01, MIAPaCa‐2 alone versus MIAPaCa‐2/other subtypes: *p* < 0.001, other comparisons: N.S. MIAPaCa‐2/subtype A versus MIAPaCa‐2/other subtype comparison, unpaired *t*‐test with Welch's correction: *p* = 0.028. MIAPaCa‐2/PS1 versus MIAPaCa‐2/subtype A and MIAPaCa‐2/PS1 versus MIAPaCa‐2/other subtype comparison, unpaired *t*‐test with Welch's correction: *p* < 0.001. (D) Representative pictures of H&E‐stained sections for cell invasion at D12 in MIAPaCa‐2/PS1 co‐cultures, MIAPaCa‐2/subtype‐A CAF and MIAPaCa‐2/other‐subtype CAF co‐cultures (*n* = 1 primary CAF culture per subtype). MIAPaCa‐2 alone: no invasion. Arrows point at invading cells. Scale bar: 50 μm. (E) Cell proliferation at D4 assessed by the ratio of Ki67‐positive nuclei over the total number of nuclei in cancer cells (PDGFRα‐negative). Mean ratio: 0.60 ± 0.05 in MIAPaCa‐2 alone (triplicate), 0.67 ± 0.05 in MIAPaCa‐2/PS1 co‐culture (triplicate), 0.74 ± 0.04 in MIAPaCa‐2/subtype‐A CAF co‐culture (*n* = 2 distinct CAF cultures) and 0.91 ± 0.05, in MIAPaCa‐2/other‐subtype CAF co‐culture (*n* = 2 distinct CAF cultures), Kruskal–Wallis: *p* = 0.0001. Dunn's multiple comparisons: MIAPaCa‐2 alone versus MIAPaCa‐2/other subtypes: *p* < 0.01, other comparisons: NS. MIAPaCa‐2/subtype A versus MIAPaCa‐2/other subtype comparison, unpaired *t*‐test with Welch's correction: *p* = 0.001. MIAPaCa‐2/PS1 versus MIAPaCa‐2/subtype A and MIAPaCa‐2/PS1 versus MIAPaCa‐2/other subtype comparison, unpaired *t*‐test with Welch's correction: *p* = 0.13 and *p* = 0.002, respectively. (F) Correlation plot between cell layer thickness and Ki67‐based proliferation. MIAPaCa‐2 monocultures are displayed in black, MIAPaCa‐2/PS1 co‐cultures in blue, MIAPaCa‐2/subtype‐A CAF co‐cultures in red and MIAPaCa‐2/other‐subtype CAF co‐cultures in grey. Spearman *r* = 0.9265, *p* < 0.0001. (G) Cell proliferation at D4 assessed by the cell layer thickness in control or gemcitabine‐treated (concentration: 100 nm = IC_50_ of MIAPaCa‐2 alone) mini‐organotypics, normalised to control in each group (triplicate for PS1 co‐culture and *n* = 2 distinct CAF cultures per subtype for primary cultures). One‐way ANOVA with Sidak's multiple comparisons, mean difference gemcitabine‐treated versus control in MIAPaCa‐2/other‐subtype CAF co‐cultures: 0.26 [0.004–0.51]; in MIAPaCa‐2/subtype‐A CAF co‐cultures: 0.58 [0.41–0.74], *p* ≤ 0.01; in MIAPaCa‐2/PS1: 0.69 [0.55–0.84], *p* ≤ 0.0001.

With this phenotypic knowledge, and bearing in mind the paucity of primary CAFs and their associated inherent propagation limitations, we compared the functional impact of pCAF subtype A versus other subtypes on cancer cells, in the well‐validated pancreatic mini‐organotypic model 28, using PS1 cell line as reference standard and cancer cell (MIAPaCa‐2 or AsPC‐1) monocultures without CAF/PSC as negative control (Figure 5A). Co‐cultures of MIAPaCa‐2 with primary CAF cultures resulted in increased cancer cell proliferation in comparison to co‐culture with non‐tumoural PSCs (PS1), as assessed by mean cell layer thickness at day 4 (*p* < 0.0001), and increased cancer cell invasion at day 12 (*p* = 0.024) (Figure 5B–D and supplementary material, Figure S5A). These results with the MIAPaCa‐2 cancer cell line were validated in co‐cultures with the AsPC‐1 cell line (see supplementary material, Figure S5B,C). Composition of CAF cultures in terms of predominant subtype A versus other subtypes translated into differential functional effects on cancer cells. Mini‐organotypic co‐cultures with CAF cultures of other subtypes induced more cancer cell proliferation (cell layer thickness) than subtype A co‐cultures (*p* = 0.028) (Figure 5B,C). Consistently, Ki67 expression by IHC in cancer cells (ratio of Ki67‐positive/total nuclei in PDGFRα‐negative cells, supplementary material, Figure S5D) was increased in mini‐organotypic co‐cultures with other pCAF subtypes (*p* = 0.001) (Figure 5E). This finding demonstrated that the increase in cell layer thickness with other pCAF subtypes was the direct consequence of cancer cell proliferation induction. Cell layer thickness and Ki67‐based proliferation were correlated (*p* < 0.0001), allowing the use of cell layer thickness as a surrogate for Ki67‐based proliferation assessment in following experiments (Figure 5F). Finally, non‐subtype A CAFs were associated with cancer cell protection against gemcitabine in mini‐organotypic co‐cultures with MIAPaCa‐2 (*p* < 0.0001) (Figure 5G and supplementary material, Figure S5E) 3. Taken together, these data suggest that pCAF subtype A CAFs may be associated with a less pro‐tumoural (less pro‐proliferative and chemoprotective to cancer cells) profile than other, non‐subtype A CAFs.

### CAF subtypes reflect dynamic PSC‐CAF evolution

**Figure 6:**
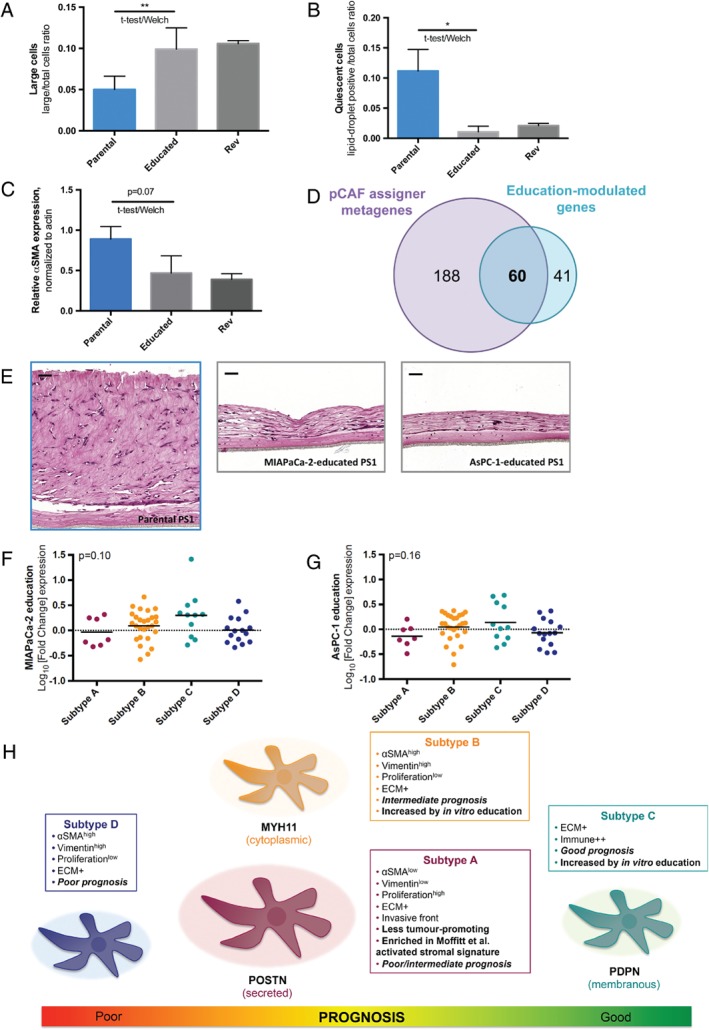
Pancreatic stellate cell (PSC)‐ cancer‐associated fibroblasts (CAF) dynamics. (A) Ratio of large cells over total cell number in parental PS1, following 2‐month education (‘Educated’), and after 1‐month wash‐out period in normal medium (reversion, ‘Rev’). Mean ratio in parental PS1 versus educated PS1 (duplicate): 0.050 ± 0.016 versus 0.099 ± 0.026, unpaired *t*‐test with Welch's correction, *p* = 0.006. (B) Ratio of lipid‐droplet‐positive (quiescent) cells over total cell number in parental PS1 upon all‐trans retinoic acid (ATRA) treatment (1 μm daily, for 5 days), following 2‐month education, and after 1‐month wash‐out period in normal medium (Rev). Mean ratio in parental PS1 versus educated PS1 (duplicate): 0.11 ± 0.04 versus 0.01 ± 0.01, unpaired *t*‐test with Welch's correction, *p* = 0.006. C Quantification of αSMA expression normalised to β‐actin (actin) using ImageJ, in parental PS1, following 2‐month education with MIAPaCa‐2 or AsPC‐1 CM, and after 1‐month wash‐out period in standard medium (Rev). Mean expression in parental PS1 versus educated PS1 (duplicate): 0.89 ± 0.16 versus 0.47 ± 0.21, unpaired *t*‐test with Welch's correction, *p* = 0.07. (D) Venn diagram showing the overlap (*n* = 60 genes) between pCAFassigner metagenes (*n* = 248) and education‐modulated genes (*n* = 101, variance <0.25 in parental PS1). (E) Representative pictures of H&E‐stained slides in mini‐organotypics with PS1 embedded in the gel in parental PS1, MIAPaCa‐2‐educated PS1, and AsPC‐1‐educated PS1. Scale bar: 50 μm. (F) Modulation (log_10_(fold‐change)) of CAF subtype‐specific genes in MIAPaCa‐2‐educated PS1 versus parental PS1. One‐way ANOVA: *p* = 0.10. (G) Modulation (log(fold‐change)) of CAF‐subtype specific genes in AsPC‐1‐educated PS1 versus parental PS1. One‐way ANOVA: *p* = 0.16. (H) Pancreatic CAF heterogeneity model. CAF subtypes were associated with distinct molecular and functional features (ECM‐ and immune‐related signatures, intra‐tumoural spatial pattern of expression, vimentin and αSMA expression, proliferation rate, tumour‐promoting and chemoprotective capabilities) and had a prognostic impact. Periostin (POSTN), myosin‐11 (MYH11) and podoplanin (PDPN) were identified as subtype A, B and C markers, respectively.

We designed a ‘cancer‐education’ experiment to explore the tumour‐stroma interaction. We exposed non‐tumoural PSCs (PS1) to conditioned media (CM) from cancer cell lines (MIAPaCa‐2 and AsPC‐1) for 2 months in standardised culture conditions (see supplementary material, Figure S6A). Following the education process, PSC cultures (1) became enriched in large cells with a clear nucleus (*p* = 0.006) (Figure 6A and supplementary material, Figure S6B), and (2) lost their sensitivity to ATRA (*p* = 0.03) (Figure 6B and supplementary material, Figure S6C), consistent with primary CAF features, suggesting a potential ‘CAF‐like’ phenotypic switch. In addition, educated PS1 displayed decreased αSMA expression (assessed by western blot, *p* = 0.07) (Figure 6C and supplementary material, Figure S6D). Moreover, these features were maintained after a 1‐month wash‐out period in standard medium (‘reversion’ samples). Overall, no significant phenotypic difference was observed between MIAPaCa‐2‐ and AsPC‐1‐education of PS1 cells *in vitro*. We next compared gene expression in educated versus parental PS1, using Nanostring analysis. Remarkably, out of the 101 genes that appeared significantly modulated by education, 60 had been previously identified in the pCAFassigner extended gene list (Figure 6D). Consistent with previous phenotypic findings, there was a large overlap in up‐/down‐regulated genes between MIAPaCa‐2‐ and AsPC‐1‐educated PS1 (Fisher's exact test, *p* < 0.0001), with only 8/60 (13.3%) genes showing opposite regulation (see supplementary material, Table S6). The 31 common genes up‐regulated in both MiAPaCa‐2‐educated and AsPC‐1‐educated PS1 (log_10_(fold change) > 0) were involved in ECM regulation (including matrix metalloproteinases), as well as immune pathways (including lymphocyte and granulocyte pathways), which was evocative of subtype C signature (see supplementary material, Table S7). Conversely, the 21 identified common down‐regulated genes (log(fold change) < 0) were involved in matrisome and core matrisome, suggesting a switch in the balance between ECM production and degradation (see supplementary material, Table S7). In mini‐organotypic models with PS1 cells seeded within the gel, there was a remarkable reduction in gel thickness with educated PS1 compared to parental PS1, thus functionally validating the RNA‐signatures associated with ‘cancer‐education’ experiments (Figure 6E).

By analysing the 60 genes into pCAF subtypes, we observed that they were differentially modulated by education (*p* < 0.0001 and *p* = 0.035 in MIAPaCa‐2‐ and AsPC‐1‐educated PS1, respectively, when considering fold change values, and *p* = 0.10 and *p* = 0.16, respectively, when considering log(fold change) values) (Figure 6F,G). The ratio of numbers of up‐regulated genes over total genes per subtype showed an increase in both subtypes B and C (ratio > 0.5), showing that transcriptomic signatures associated with these two CAF subtypes can be induced by exposure to soluble factors (see supplementary material, Figure S6E,F).

Immunofluorescence co‐staining experiments with POSTN, MYH11, and PDPN on parental and educated PS1 (see supplementary material, Figure S6G) revealed that while POSTN was diffusely expressed in PS1 across all the conditions, MIAPaCa‐2 and AsPC‐1‐educated PS1 showed a homogeneous decrease in POSTN (subtype A‐related) staining intensity and increase in MYH11‐positive (subtype B‐related) and PDPN‐positive (subtype C‐related) cells, with presence of double‐ and triple‐positive hybrid cells, suggesting for the first time (to our knowledge) an *in vitro* read‐out for cancer‐educated fibroblasts. This switch in pCAFassigner marker expression in educated PS1 might represent a transition from subtype A‐like phenotype toward subtype B/C.

## Discussion

Using molecular and functional analyses on human PDAC‐derived CAF primary cultures, as well as *in silico* and IHC analyses, we propose a classification of pancreatic CAFs (pCAFassigner), demonstrating inter‐ and intra‐tumoural heterogeneity of CAFs in human PDAC. We identified at least four distinct pCAF subtypes, associated with specific phenotypic features and prognostic impact. Periostin (a subtype A biomarker) is strongly expressed at the invasive front in human PDAC samples 24 and has been linked to tumour capsule formation at the primary tumour site 29 and metastatic niche preparation at distant sites 30, 31. Furthermore, high POSTN protein expression was associated with aggressive molecular tumour features and shorter survival. Lastly, our *in vitro* functional data demonstrates that subtype A is less pro‐tumoural, suggesting that this subtype may be more a consequence than a cause of aggressive PDAC behaviour.

Myosin‐11, a smooth muscle myosin belonging to the myosin heavy chain family, was selected as subtype B marker. Interestingly, Lambrechts *et al* 16 identified a cluster (fibroblast 2) expressing αSMA and MYH11, displaying myogenic properties, similar to pCAF subtype B. Podoplanin‐positive CAFs have been previously associated with poor prognosis in several cancers 32, 33, 34, 35, 36, 37. In PDAC, PDPN expression was associated with larger tumours 36, and was relevant for prognosis only in large tumours with lymph node metastasis 37, which themselves are adverse prognostic features. Subtype C CAFs (where PDPN is one of the top genes) appear to have an immunogenic profile, which, in part, may explain the good prognosis observed in the ICGC and IHC analysis. Some studies in other cancer types support this hypothesis, showing a positive association between PDPN‐positive CAFs and lymphocyte and macrophage infiltration 38, 39, 40, as well as tumours with a high mutation burden 41, 42, suggesting that PDPN expression may be an indicator of immunogenic tumours. We summarise these findings in Figure 6H.

Moreover, our cancer education experiment showed that several pCAF subtypes can be induced from PSCs *in vitro*, and suggested that CAF subtypes might be dynamic, fluctuating states for CAFs which may be modulated by signals from cancer cells, but also possibly by other stromal cells, such as immune cells. An alternative hypothesis is that CAF subpopulations may emerge from distinct cellular origins 43, 44.

This classification was achieved through international collaboration. We believe that it merits independent, prospective validation of the proposed inter‐ and intra‐tumoural heterogeneity, dynamics and prognostic impact in larger, independent cohorts, and also the evaluation of its relevance to other pancreatic diseases, such as chronic pancreatitis, as well as functional ascertainment in murine models of PDAC. Single cell analysis 16, 45 may further advance the understanding of CAF heterogeneity that is currently suggested by our bulk culture analyses, in which there is a mixture of intra‐tumour heterogeneous CAFs. In addition, this technique may reveal additional subtypes that cannot be cultured or expanded.

So far, studies of CAF subpopulations in PDAC and other cancers have been mainly descriptive and relied on previously reported stromal markers 6, 7, 46, 47, 48. Although IHC analyses, using multiple CAF markers, confirmed the positivity of most PDAC tumour samples for these proteins, these authors did not demonstrate the simultaneous existence of spatially distinct CAF subpopulations (i.e. intra‐tumour heterogeneity) and did not explore their functions 46. Ikenaga *et al* 47 showed the presence of two subpopulations of CAFs in human PDAC stroma (CD10‐positive and CD10‐negative), with the former having a more pro‐tumoural role. Su *et al* 49 recently confirmed this finding in breast and lung cancers, and demonstrated that a subset of CD10‐positive CAFs (CD10+GPR77+) promote cancer formation and chemoresistance by sustaining cancer stemness. In contrast, we used a ‘without *a priori*’ approach, based on the transcriptomic profile of primary cultures to build an assignation, and described distinct phenotypic profiles of CAF subpopulations. Öhlund *et al* 7 proposed a binary, simultaneous existence of αSMA‐positive (‘myofibroblastic CAFs’), and αSMA‐negative/IL6‐positive (‘inflammatory CAFs’) subpopulations in spatially distinct zones in PDAC tissue, mainly through murine data. Kalluri *et al* 17 also presented preliminary data for a binary classification based on FAP and αSMA expression. Using genetic ablation of FAP‐positive or αSMA‐positive CAF populations in mouse models, they identified FAP‐positive CAFs as pro‐tumoural versus αSMA‐positive CAFs as anti‐tumoural. Our results indicate that CAF heterogeneity in PDAC is more complex than a “αSMA‐positive versus negative” dichotomy.

Intriguingly, it has been suggested that CAFs grown *ex vivo* as monolayer cultures should converge towards a singular myofibroblastic, αSMA‐positive profile 7. This is refuted by our observation that both αSMA‐positive and ‐negative CAFs can be successfully grown in monolayer culture conditions. In addition, it has been reported that cancer cells, *in vitro* 7 and *in vivo* 50, could quickly recruit and subvert non‐tumoural PSCs to a phenotype that aids cancer cell growth and metastasis. We showed that, beyond transitory activation, a stable ‘CAF‐like’ phenotype, including loss of capacity to revert to quiescence, can be induced by prolonged exposure of non‐tumoural PSCs to CM from cancer cell lines *in vitro* (cancer‐education and reversibility experiments), providing new insight into PSC/CAF plasticity 6, 50.

Our demonstration that PDAC CAFs are not a homogenous entity may partially account for inconsistencies in preclinical results and the failure of some stroma‐targeting agents 8, 9, 10. Caution is thus indicated when interpreting results from experimental models using immortalised CAFs or primary cultures. Indeed, we showed that pCAFs display molecular and functional diversity, like cancer cells and other immune cells, and they should be carefully characterised. We postulate that our molecular classification and derived assays will allow better understanding of PDAC tumour‐stroma interactions.

Deciphering PDAC heterogeneity is a major goal to improve therapeutic strategies and patient management. As CAFs play a crucial role as microenvironment orchestrators, particularly by producing ECM and interacting with cancer and immune cells 6, 51, they are involved in PDAC therapeutic resistance. Our results provide the first evidence for CAF‐based patient prognostic stratification in PDAC. In breast cancer, CAF subtypes have already been proposed as predictive markers of response to immune therapy 52. It is therefore envisaged that therapeutic advantage may be gained by specifically targeting deleterious, immunosuppressive CAF subpopulations 53.

## Author contributions statement

CN, AT‐R and HK contributed to the original idea. CN, AT‐R, AS and HK contributed to the design of the work. CN, AT‐R, CR, JC, MM, ASW and FRD contributed to the acquisition of data. CN, YP, MT, AT‐R, JC, AS and HK contributed to the analysis/interpretation of data. ME, JK, JW, MA, JC, AS and HK contributed to the provision of material. CB, PH, VP, AS and HK contributed to the study supervision. CN, AS and HK contributed to the manuscript writing. All the authors approved the manuscript revision.

**SUPPLEMENTARY MATERIAL ONLINE**

**Supplementary methods**

**Supplementary figure legends**

**Figure S1.** Classification of PDAC CAF

**Figure S2.** Validation of pCAFassigner

**Figure S3.** Intra‐tumoural CAF heterogeneity in human PDAC

**Figure S4.** Phenotypic features of CAF

**Figure S5.** Differential influence of CAF subtypes on cancer cells

**Figure S6**. Phenotypic modulation of CAF subtypes

**Table S1.** Clinico‐pathological characteristics of the 16 tumours used for primary CAF culture isolation

**Table S2.** *KRAS* mutation status

**Table S3.** pCAFassigner subtype assignment in the ICGC dataset

**Table S4.** Summary of ICGC sample distribution according to first and second CAF subtypes

**Table S5.** Classification of the 50 evaluable samples (IHC cohort) based on POSTN, MYH11 and PDPN expression levels

**Table S6.** Contingency table of up‐regulated or down‐regulated genes following education of MIAPaCa‐2 or AsPC‐1 cells

**Table S7.** Gene expression pathway analyses in *in vitro* educated PS1 cells

**Table S8.** Culture media and conditions for cell lines

**Table S9.** Antibodies used for western blotting

**Table S10.** Antibodies used for immunofluorescence

**Table S11.** Antibodies used for immunohistochemistry

## Supporting information


**Supplementary methods**

**Supplementary figure legends**

**Figure S1.** Classification of PDAC CAF. (A) Illustrative example of pyrosequencing results for *KRAS* codon 12 in AsPC‐1 (mutated), PS1 (WT), two CAF primary cultures (WT, PSC25 and PSC11) and control DNA. (B) Principal component analysis (PCA) plot of CAF primary cultures before batch correction. (C) PCA plot of CAF primary cultures after batch correction. (D) Silhouette plot for *k* = 2 to *k* = 5 classes. (E) Consensus matrices for *k* = 2 to *k* = 5 classes

**Figure S2.** Validation of pCAFassigner. (A) Cophenetic correlation, silhouette plot and consensus matrix clustering for standard deviation (SD) cut‐off of 1.5. (B) Cophenetic correlation, silhouette plot and consensus matrix clustering for SD = 1.2. (C) Cophenetic correlation, silhouette plot and consensus matrix clustering for SD = 0.8. (D) Heat map with hierarchical clustering for the 248 metagenes that were found differentially expressed between patient‐derived CAF primary cultures (extended pCAFassigner). Significantly higher expression is shown in red and lower expression in green

**Figure S3.** Intra‐tumoural CAF heterogeneity in human PDAC. (A) Expression of pCAFassigner subtype‐specific genes that express similar to that of the correlation plot (Figure 2B) and are associated with Lambrechts fibroblast subtypes 16. (B) H&E stain and IHC for periostin (POSTN), myosin‐11 (MYH11), podoplanin (PDPN), αSMA and PDGFRα on serial sections from a second patient‐derived resected PDAC sample. Low magnification: scale bar: 1 mm; high magnification (inset): scale bar: 100 μm. (C) Immunofluorescence co‐staining of POSTN (green), MYH11 (red or green), PDPN (red) and DAPI (blue) on PSC25 (subtype A), PSC48 (mixed, subtype A dominant > B) and PSC11 (subtype C). Percentages of positive cells for each marker are displayed. Scale bar: 200 μm. (D) Association between CAF subtypes and OS in the Moffitt *et al* normal stroma group from the ICGC dataset (*n* = 26). Log‐rank test, *p* = 0.14. Median OS in subtype A/B: 15.9 months, subtype C: 50.4 months and subtype D: 21.0 months.

**Figure S4.** Phenotypic features of CAF. (A) CAF subtype‐specific expression of αSMA by western blot, quantified using ImageJ and normalised to PS1 (*n* = 16). Kruskal–Wallis test, *p* = 0.051. (B) CAF subtype‐specific expression of vimentin by western blot, quantified using ImageJ and normalised to PS1 (*n* = 16). Kruskal–Wallis test, *p* = 0.016. (C) CAF subtype‐specific expression of platelet‐derived growth factor receptor α (PDGFRα) by western blot, quantified using ImageJ and normalised to PS1 (*n* = 16). Kruskal–Wallis test, *p* = 0.37. (D) αSMA (green), vimentin (red) expression and DAPI (blue) by immunofluorescence in PS1, M1090 T (αSMA‐low by western blot) and M1198 (αSMA‐high by western blot). Scale bar: 50 μm. (E) Correlation between αSMA high/low expression status based on western blot and immunofluorescence (*n* = 10). (F) Proliferation curves assessed by MTS assay in CAF primary cultures. Subtype A CAFs are displayed in red and other subtypes in grey (*n* = 16). (G) CAF subtype‐specific area under the proliferation curve (AUC) by MTS, normalised to PS1 (*n* = 16). Kruskal–Wallis test, *p* = 0.051. (H) CAF subtype‐specific ratio of lipid‐droplet‐positive cells over total cells, assessed by Oil Red O staining, in all‐trans retinoic acid (ATRA)‐treated CAF primary cultures (*n* = 16). Kruskal–Wallis test, *p* = 0.99. (I) Cell surface area measured using ImageJ, according to pCAFassigner subtype (total: *n* = 5, subtype A: *n* = 2, subtype C: *n* = 1, subtype D: *n* = 2, 10 cells per CAF culture). Mean cell surface normalised to PS1: 1.00 ± 0.89 in PS1, 4.05 ± 2.00 in subtype‐A CAF, 4.45 ± 3.10 in other‐subtype CAF, Kruskal–Wallis test, *p* < 0.001. Dunn's multiple comparisons: PS1 versus subtype A: *p* < 0.001, PS1 versus other subtypes: *p* < 0.001, subtype A versus other subtypes: NS.

**Figure S5.** Differential influence of CAF subtypes on cancer cells. (A) Quantification of invading cells in gels using ImageJ. Mean number of invading cells per gel: 14.7 ± 7.4 and 360.8 ± 97.1 in MIAPaCa‐2/PS1 and in MIAPaCa‐2/CAF co‐cultures, respectively. Unpaired *t*‐test with Welch's correction: *p* = 0.024. (B) Representative pictures of H&E‐stained sections of mini‐organotypics for cell proliferation at day 4 (D4) in AsPC‐1 monoculture, and AsPC‐1/PS1 and AsPC‐1/subtype‐A CAF co‐cultures. Scale bar: 50 μm. (C) Representative pictures of H&E‐stained sections of mini‐organotypics for cell invasion at D12 in AsPC‐1/PS1 and AsPC‐1/subtype‐A CAF co‐cultures. AsPC‐1 alone: no invasion. Scale bar: 50 μm. (D) Representative pictures of Ki67 (brown) and PDGFRα (red) co‐immunostaining in MIAPaCa‐2 monoculture and in MIAPaCa‐2/PSC25 (subtype A) co‐culture. Scale bar: 100 μm. (E) Representative pictures of H&E‐stained sections of control or gemcitabine (100 nm)‐treated mini‐organotypics at D4 in MIAPaCa‐2 monoculture, MIAPaCa‐2/PS1, MIAPaCa‐2/PSC25 (subtype A) CAF and MIAPaCa‐2/PSC11 (other subtype) CAF co‐cultures. The concentration of 100 nm was selected as the IC_50_ of gemcitabine in MIAPaCa‐2 3D monocultures. Black lines highlight cell layer thickness. Scale bar: 100 μm.

**Figure S6.** Phenotypic modulation of CAF subtypes. (A) Overview of the ‘education’ experiment design. Immortalised pancreatic stellate cells (PSCs, PS1 cell line) were exposed to conditioned media (CM) from either MIAPaCa‐2 or AsPC‐1 cancer cell lines for 2 months and then put back in standard medium for 1 month (reversion, Rev). This experiment was performed twice. (B) Representative bright‐field pictures of parental and educated PS1 (MIAPaCa‐2‐educated, MIA‐ed. and AsPC‐1‐educated, ASPC1‐ed.). Arrows point at large cells. Scale bar: 50 μm. (C) Representative pictures of all‐trans retinoic acid (ATRA)‐treated parental and educated PS1 after Oil Red O staining (MIAPaCa‐2‐educated, MIA‐ed. and AsPC‐1‐educated, ASPC1‐ed.). Arrows point at lipid‐droplet‐positive cells. Scale bar: 50 μm. (D) αSMA, vimentin and β‐actin (actin) expression in parental PS1, following 2‐month educated (MIA‐ed. and ASPC1‐ed.), and after 1‐month wash‐out period in standard medium (Rev MIA‐ed. and Rev ASPC1‐ed.) by western blot. Parental PS1 are displayed in blue, educated PS1 in light grey and Rev samples in dark grey. #1 and #2 refer to the batch number. (E) Distribution of up‐regulated versus down‐regulated genes according to CAF subtypes in MIAPaCa‐2‐educated PS1. Ratio of numbers of up‐regulated genes over total genes for subtypes B and C: 19/27 and 8/11, respectively. Chi‐square *P* value: 0.27. (F) Distribution of up‐regulated versus down‐regulated genes according to CAF subtypes in AsPC‐1‐ educated PS1. Ratio of numbers of up‐regulated genes over total genes for subtypes B and C: 18/27 and 7/11, respectively. Chi‐square *P* value: 0.16. (G) Immunofluorescence co‐staining of POSTN (green), MYH11 (red or green), PDPN (red) and DAPI (blue) on parental and MIAPaCa‐2‐ or AsPC‐1‐educated PS1 (MIA‐ed. and ASPC1‐ed.). Scale bar: 200 μm.

**Table S1.** Clinico‐pathological characteristics of the 16 tumours used for primary CAF culture isolation
**Table S2.** *KRAS* mutation status
**Table S3.** pCAFassigner subtype assignment in the ICGC dataset
**Table S4.** Summary of ICGC sample distribution according to first and second CAF subtypes
**Table S5.** Classification of the 50 evaluable samples (IHC cohort) based on POSTN, MYH11 and PDPN expression levels
**Table S6.** Contingency table of up‐regulated or down‐regulated genes following education of MIAPaCa‐2 or AsPC‐1 cells
**Table S7.** Gene expression pathway analyses in *in vitro* educated PS1 cells
**Table S8.** Culture media and conditions for cell lines
**Table S9.** Antibodies used for western blotting
**Table S10.** Antibodies used for immunofluorescence
**Table S11.** Antibodies used for immunohistochemistry
