# Supplementary material for: Inter‐ and intra‐tumoural heterogeneity in cancer‐associated fibroblasts of human pancreatic ductal adenocarcinoma
Source: J Pathol. 2019 Feb 22;248(1):51–65. doi: 10.1002/path.5224 (PMC6492001; doi:10.1002/path.5224)
Supplement: Supplementary file 1 — Supplementary methods [file PATH-248-51-s001.docx]

**SUPPLEMENTARY METHODS**

**Cell culture**

The MIAPaCa-2 and AsPC-1 human PDAC cell lines and the MRC5 human embryonic lung fibroblasts were obtained from the American Type Culture Collection (ATCC, Rockville, USA). The PS1 cell line (immortalised non-tumoural PSC) was developed and characterized previously [26]. An ATCC short tandem repeat (STR) profile certification was obtained for each cell line. Detailed culture media and conditions are provided in Table S8. Cells were checked for the absence of mycoplasma contamination using nested polymerase chain reaction (PCR) assay.

**Mini-organotypic experimental system (see Figure 5A)**

Briefly, either cancer cells alone (control) or cancer cells admixed with PS1 or primary CAF single culture in a 1:2 ratio, were plated onto gels composed of 75% collagen type I (3 mg/ml, 5.25 volumes) and 25% Matrigel (1.75 volumes; both Corning, Corning, NY, USA), 1 volume of 10 × DMEM, 1 volume of 1 × DMEM, and 1 volume of filtered FBS, in 6.5-mm permeable inserts with 0.4-μm pores (Corning, Corning, NY, USA). The next day, culture medium was removed and replaced by fresh medium with 10% FBS without drug, or with 100 nM gemcitabine (Eli Lilly, Indianapolis, IN, USA) (concentration corresponding to MIAPaCa-2 3D IC50). Mini-organotypic cultures were incubated for 4 (proliferation time point, day 4 [D4]) to 12 days (invasion time point, day 12 [D12]) at 37°C/5% CO2. Medium with FBS (below the insert) was replaced and gels were rehydrated using medium without FBS (at the top) every 2 days from day 4. At the end of the experiment, the gels were harvested, fixed in 10% neutral buffered formalin, then submerged in 70% ethanol, bisected, and embedded in paraffin.

**Nucleic acid extraction and analysis**

DNA was extracted using AllPrep® DNA/RNA MiniKit and QiaCube (Qiagen, Hilden, Germany), according to the manufacturer’s instructions, and quantified using Nanodrop. Mutations in *KRAS* codons 12/13 (G12A, G12C, G12D, G12R, G12S, G12V, G13D) and 61 (Q61E, Q61H, Q61L, Q61R) were screened by pyrosequencing using Therascreen Pyro Kit (Qiagen, Hilden, Germany). mRNA was prepared using AllPrep® DNA/RNA MiniKit (Qiagen, Hilden, Germany) and quantified using Nanodrop.

***In vitro* assays**

Western blots, immunofluorescence, MTS (3-(4,5-dimethylthiazol-2-yl)-5-(3-carboxymethoxyphenyl)-2-(4-sulfophenyl)-2H-tetrazolium, inner salt) assays, ATRA treatment, and mini-organotypic cultures were carried out as described before [55]. Antibody details are provided in Tables S9 and S10. For immunofluorescence co-staining studies, double stainings with pairs of primary antibodies (POSTN/MYH11, POSTN/PDPN, MYH11/PDPN) were performed. Negative controls were primary antibodies alone, and secondary antibodies alone (no staining observed).

**Beaujon Hospital patient cohort**

Fifty patients were selected from a retrospective cohort of patients with localised (non-metastatic) PDAC who underwent complete surgical resection between December 2011 and January 2014 at Beaujon University Hospital (Clichy, France; biobank registration number BB-0033-00078). All participants gave informed consent before taking part. Exclusion criteria were: neoadjuvant chemotherapy and/or radiotherapy, macroscopically incomplete resection (R2), tumour histology other than PDAC, and insufficient tumour material available for research. Overall survival (OS) was defined as the time interval between the day of surgical resection and death or the date of the last follow-up, at which point data were censored. Furthermore, one patient who died within 30 days following surgery was excluded from survival analysis.

**Immunohistochemistry**

Entire tumour sections were obtained from formalin-fixed, paraffin-embedded tumour specimens. Pathological data were checked on H&E-stained tumour section slides, and one representative block was selected. All pathological slides were examined by a pathologist with expertise in pancreatic tumours (JC). Immunostainings (Table S11) were performed on an automated platform (Ventana Benchmark®). Slides were scanned using a computer-controlled capture device (AT turbo, Aperio®; Leica Biosystems, Nanterre, France) and quantified by ImageJ® software. Tumour section slides were examined and scored visually by two observers (JC and CN). Internal positive controls were smooth muscle in vessel and bowel wall for MYH11, and nerves and lymphatics for PDPN. High periostin expression was defined as moderate or strong staining in >50% of stromal surface. High myosin-11 (MYH11) and podoplanin (PDPN) expressions were defined as the presence of strong stromal staining. In case of simultaneous high expression of MYH11 and PDPN, the tumour was classified according to the most abundant sub-population.

**Pathway and nearest template prediction (NTP) analyses**

Pathway analysis was performed using hypergeometric test from molecular signature database (mSigDB) database [54]. NTP was performed to predict subtypes from Moffitt *et al.* [13] using the ICGC dataset.

**Comparison of data to single fibroblast cell sequencing data**

Average gene expression data (from their supplementary information) for different fibroblast clusters from Lambrechts *et al.* [16], after median-centring genes across fibroblast clusters, were compared to pCAFassigner PAM centroids using Pearson correlation analysis.
