# Supplementary material for: Inter‐ and intra‐tumoural heterogeneity in cancer‐associated fibroblasts of human pancreatic ductal adenocarcinoma
Source: J Pathol. 2019 Feb 22;248(1):51–65. doi: 10.1002/path.5224 (PMC6492001; doi:10.1002/path.5224)
Supplement: Supplementary file 2 — Supplementary figure legends [file PATH-248-51-s009.docx]

**SUPPLEMENTARY FIGURE LEGENDS**

**Figure S1.** Classification of pancreatic ductal adenocarcinoma (PDAC) cancer-associated fibroblasts (CAF). (A) Illustrative example of pyrosequencing results for *KRAS* codon 12 in AsPC-1 (mutated), PS1 (wild-type, WT), two CAF primary cultures (WT, PSC25 and PSC11), and control DNA. (B) Principal component analysis (PCA) plot of CAF primary cultures before batch correction. (C) PCA plot of CAF primary cultures after batch correction. (D) Silhouette plot for k = 2 to k = 5 classes. (E) Consensus matrices for k = 2 to k = 5 classes.

**Figure S2.** Validation of pCAFassigner. (A) Cophenetic correlation, silhouette plot, and consensus matrix clustering for standard deviation (SD) cut-off of 1.5. (B) Cophenetic correlation, silhouette plot, and consensus matrix clustering for SD = 1.2. (C) Cophenetic correlation, silhouette plot, and consensus matrix clustering for SD = 0.8. (D) Heat map with hierarchical clustering for the 248 metagenes that were found differentially expressed between patient-derived cancer-associated fibroblast (CAF) primary cultures (extended pCAFassigner). Significantly higher expression is shown in red and lower expression in green.

**Figure S3.** Intra-tumoural cancer-associated fibroblast (CAF) heterogeneity in human pancreatic ductal adenocarcinoma (PDAC). (A) Expression of pCAFassigner subtype-specific genes that express similar to that of the correlation plot (Figure 2B) and are associated with Lambrechts fibroblast subtypes [16]. (B) H&E stain and IHC for periostin (POSTN), myosin-11 (MYH11), podoplanin (PDPN), α-smooth muscle actin (αSMA), and platelet-derived growth factor receptor α (PDGFRα) on serial sections from a second patient-derived resected PDAC sample. Low magnification: scale bar: 1 mm; high magnification (inset): scale bar: 100 μm. (C) Immunofluorescence co-staining of POSTN (green), MYH11 (red or green), PDPN (red), and DAPI (blue) on PSC25 (subtype A), PSC48 (mixed, subtype A dominant > B) and PSC11 (subtype C). Percentages of positive cells for each marker are displayed. Scale bar: 200 μm. (D) Association between CAF subtypes and overall survival (OS) in the Moffitt *et al.* normal stroma group from the ICGC dataset (n = 26). Log-rank test, p = 0.14. Median OS in subtype A/B: 15.9 months, subtype C: 50.4 months, and subtype D: 21.0 months.

**Figure S4.** Phenotypic features of cancer-associated fibroblasts (CAF). (A) CAF subtype-specific expression of α-smooth muscle actin (αSMA) by western blot, quantified using ImageJ® and normalized to PS1 (n = 16). Kruskal-Wallis test, p = 0.051. (B) CAF subtype-specific expression of vimentin by western blot, quantified using ImageJ® and normalized to PS1 (n = 16). Kruskal-Wallis test, p = 0.016. (C) CAF subtype-specific expression of platelet-derived growth factor receptor α (PDGFRα) by western blot, quantified using ImageJ® and normalized to PS1 (n = 16). Kruskal-Wallis test, p = 0.37. (D) αSMA (green), vimentin (red) expression and DAPI (blue) by immunofluorescence in PS1, M1090T (αSMA-low by western blot) and M1198 (αSMA-high by western blot). Scale bar: 50 μm. (E) Correlation between αSMA high/low expression status based on western blot and immunofluorescence (n = 10). (F) Proliferation curves assessed by MTS assay in CAF primary cultures. Subtype A CAFs are displayed in red and other subtypes in grey (n = 16). (G) CAF subtype-specific area under the proliferation curve (AUC) by MTS, normalized to PS1 (n = 16). Kruskal-Wallis test, p = 0.051. (H) CAF subtype-specific ratio of lipid-droplet-positive cells over total cells, assessed by Oil Red O staining, in all-trans retinoic acid (ATRA)-treated CAF primary cultures (n = 16). Kruskal-Wallis test, p = 0.99. (I) Cell surface area measured using ImageJ®, according to pCAFassigner subtype (total: n = 5, subtype A: n = 2, subtype C: n = 1, subtype D: n = 2, 10 cells per CAF culture). Mean cell surface normalized to PS1: 1.00 ± 0.89 in PS1, 4.05 ± 2.00 in subtype-A CAF, 4.45 ± 3.10 in other-subtype CAF, Kruskal-Wallis test, p < 0.001. Dunn’s multiple comparisons: PS1 vs. subtype A: p < 0.001, PS1 vs. other subtypes: p < 0.001, subtype A vs. other subtypes: NS.

**Figure S5.** Differential influence of cancer-associated fibroblast (CAF) subtypes on cancer cells. (A) Quantification of invading cells in gels using ImageJ®. Mean number of invading cells per gel: 14.7 ± 7.4 and 360.8 ± 97.1 in MIAPaCa-2/PS1 and in MIAPaCa-2/CAF co-cultures, respectively. Unpaired t-test with Welch’s correction: p = 0.024. (B) Representative pictures of H&E-stained sections of mini-organotypics for cell proliferation at day 4 (D4) in AsPC-1 monoculture, and AsPC-1/PS1 and AsPC-1/subtype-A CAF co-cultures. Scale bar: 50 μm. (C) Representative pictures of H&E-stained sections of mini-organotypics for cell invasion at D12 in AsPC-1/PS1 and AsPC-1/subtype-A CAF co-cultures. AsPC-1 alone: no invasion. Scale bar: 50 μm. (D) Representative pictures of Ki67 (brown) and PDGFRα (red) co-immunostaining in MIAPaCa-2 monoculture and in MIAPaCa-2/PSC25 (subtype A) co-culture. Scale bar: 100 μm. (E) Representative pictures of H&E-stained sections of control or gemcitabine (100 nM)-treated mini-organotypics at D4 in MIAPaCa-2 monoculture, MIAPaCa-2/PS1, MIAPaCa-2/PSC25 (subtype A) CAF and MIAPaCa-2/PSC11 (other subtype) CAF co-cultures. The concentration of 100 nM was selected as the IC_50_ of gemcitabine in MIAPaCa-2 3D monocultures. Black lines highlight cell layer thickness. Scale bar: 100 μm.

**Figure S6.** Phenotypic modulation of cancer-associated fibroblast (CAF) subtypes. (A) Overview of the ‘education’ experiment design. Immortalised pancreatic stellate cells (PSCs, PS1 cell line) were exposed to conditioned media (CM) from either MIAPaCa-2 or AsPC-1 cancer cell lines for two months and then put back in standard medium for 1 month (reversion, Rev). This experiment was performed twice. (B) Representative bright-field pictures of parental and educated PS1 (MIAPaCa-2-educated, MIA-ed. and AsPC-1-educated, ASPC1-ed.). Arrows point at large cells. Scale bar: 50 μm. (C) Representative pictures of all-trans retinoic acid (ATRA)-treated parental and educated PS1 after Oil Red O staining (MIAPaCa-2-educated, MIA-ed. and AsPC-1-educated, ASPC1-ed.). Arrows point at lipid-droplet-positive cells. Scale bar: 50 μm. (D) α-smooth muscle actin (αSMA), vimentin, and β-actin (actin) expression in parental PS1, following two-month educated (MIA-ed. and ASPC1-ed.), and after one-month wash-out period in standard medium (Rev MIA-ed. and Rev ASPC1-ed.) by western blot. Parental PS1 are displayed in blue, educated PS1 in light grey and Rev samples in dark grey. #1 and #2 refer to the batch number. (E) Distribution of upregulated vs. downregulated genes according to CAF subtypes in MIAPaCa-2-educated PS1. Ratio of numbers of upregulated genes over total genes for subtypes B and C: 19/27 and 8/11, respectively. Chi-square p-value: 0.27. (F) Distribution of upregulated vs. downregulated genes according to CAF subtypes in AsPC-1- educated PS1. Ratio of numbers of upregulated genes over total genes for subtypes B and C: 18/27 and 7/11, respectively. Chi-square p-value: 0.16. (G) Immunofluorescence co-staining of POSTN (green), MYH11 (red or green), PDPN (red), and DAPI (blue) on parental and MIAPaCa-2- or AsPC-1-educated PS1 (MIA-ed. and ASPC1-ed.). Scale bar: 200 μm.
