# Supplementary figures and images for: Inter‐ and intra‐tumoural heterogeneity in cancer‐associated fibroblasts of human pancreatic ductal adenocarcinoma
Source: J Pathol. 2019 Feb 22;248(1):51–65. doi: 10.1002/path.5224 (PMC6492001; doi:10.1002/path.5224)

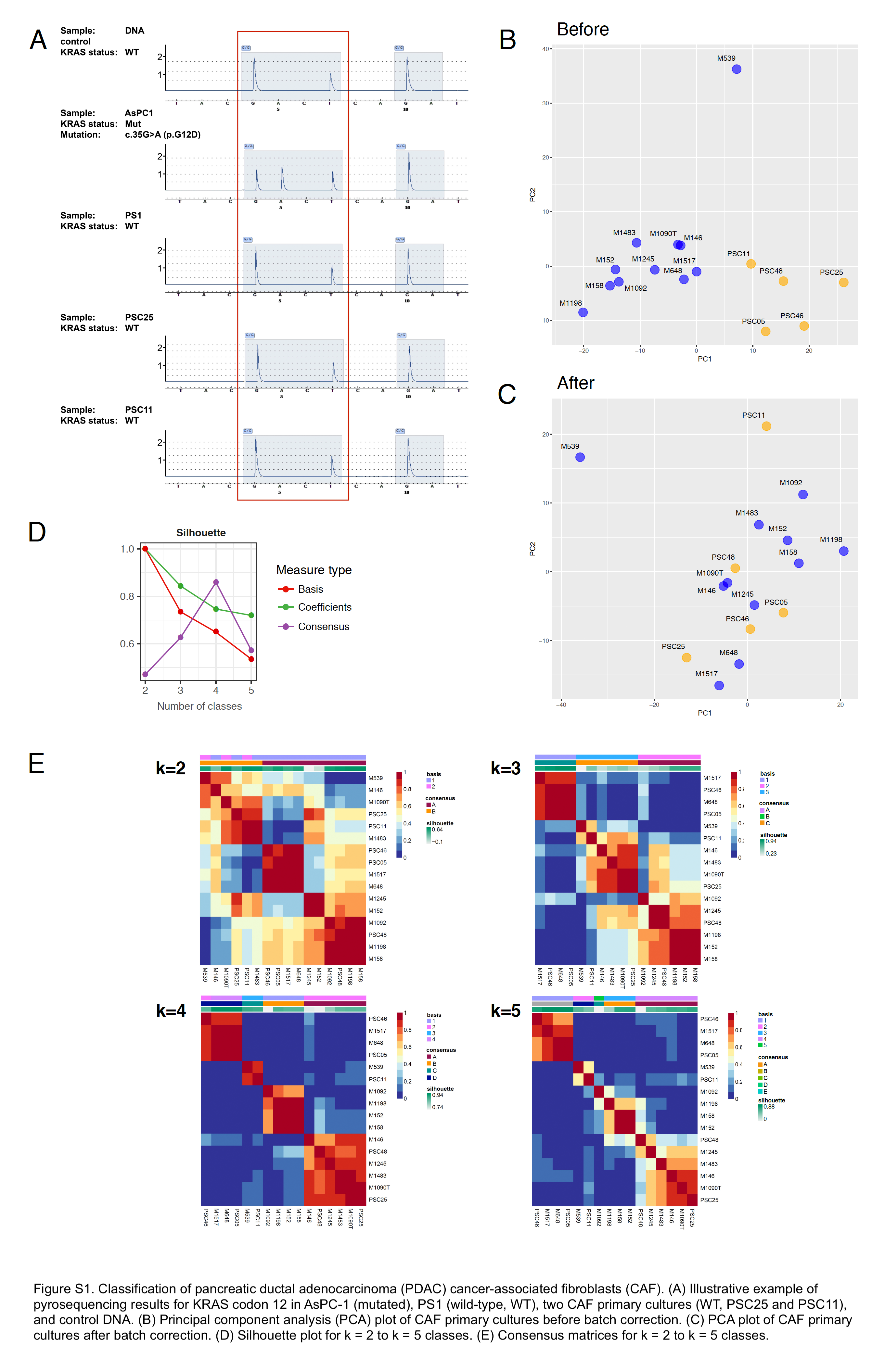

Supplement: Supplementary file 3 — Figure S1. Classification of PDAC CAF. (A) Illustrative example of pyrosequencing results for KRAS codon 12 in AsPC‐1 (mutated), PS1 (WT), two CAF primary cultures (WT, PSC25 and PSC11) and control DNA. (B) Principal component analysis (PCA) plot of CAF primary cultures before batch correction. (C) PCA plot of CAF primary cultures after batch correction. (D) Silhouette plot for k = 2 to k = 5 classes. (E) Consensus matrices for k = 2 to k = 5 classes [file PATH-248-51-s003.tif]

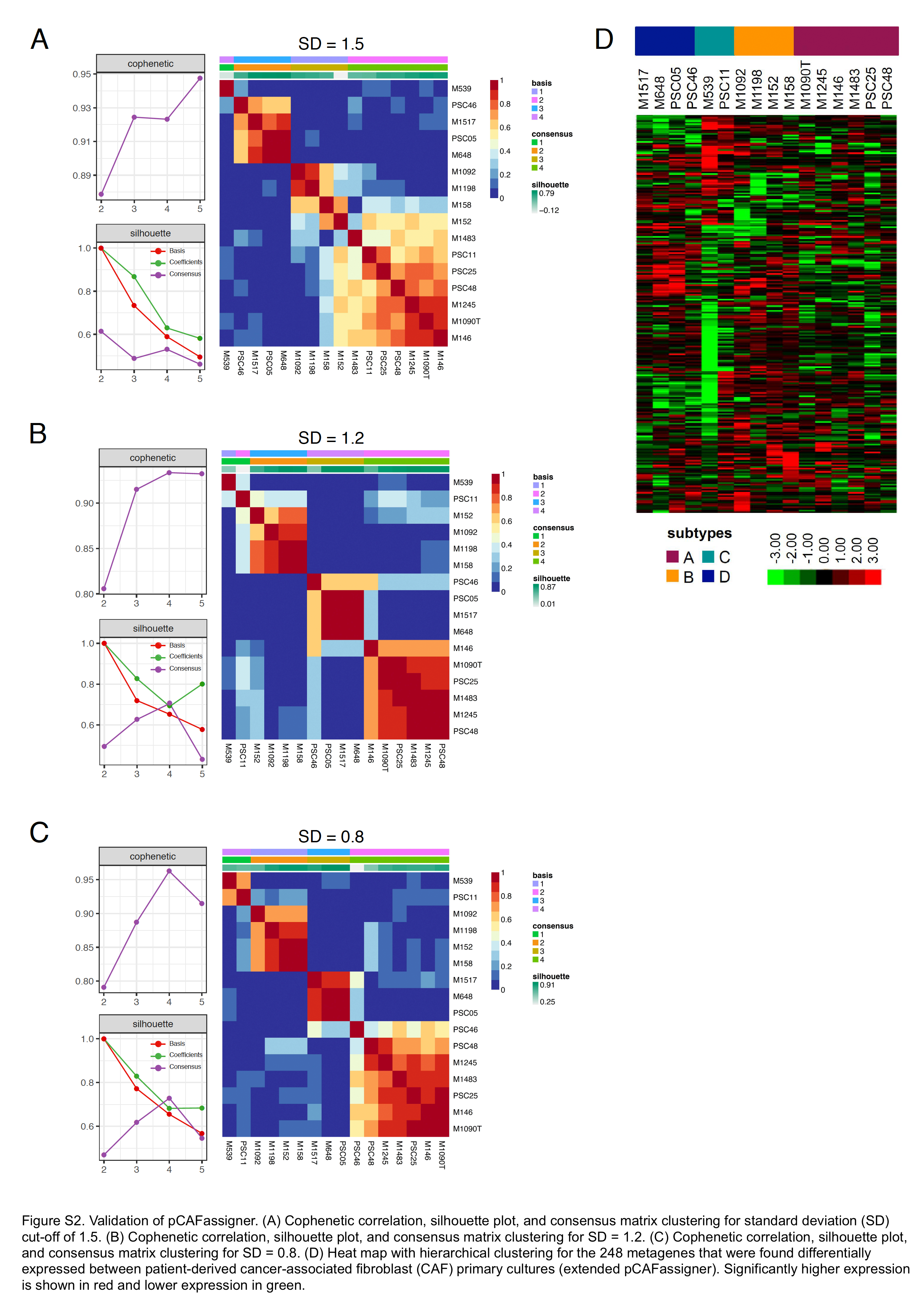

Supplement: Supplementary file 4 — Figure S2. Validation of pCAFassigner. (A) Cophenetic correlation, silhouette plot and consensus matrix clustering for standard deviation (SD) cut‐off of 1.5. (B) Cophenetic correlation, silhouette plot and consensus matrix clustering for SD = 1.2. (C) Cophenetic correlation, silhouette plot and consensus matrix clustering for SD = 0.8. (D) Heat map with hierarchical clustering for the 248 metagenes that were found differentially expressed between patient‐derived CAF primary cultures (extended pCAFassigner). Significantly higher expression is shown in red and lower expression in green [file PATH-248-51-s004.tif]

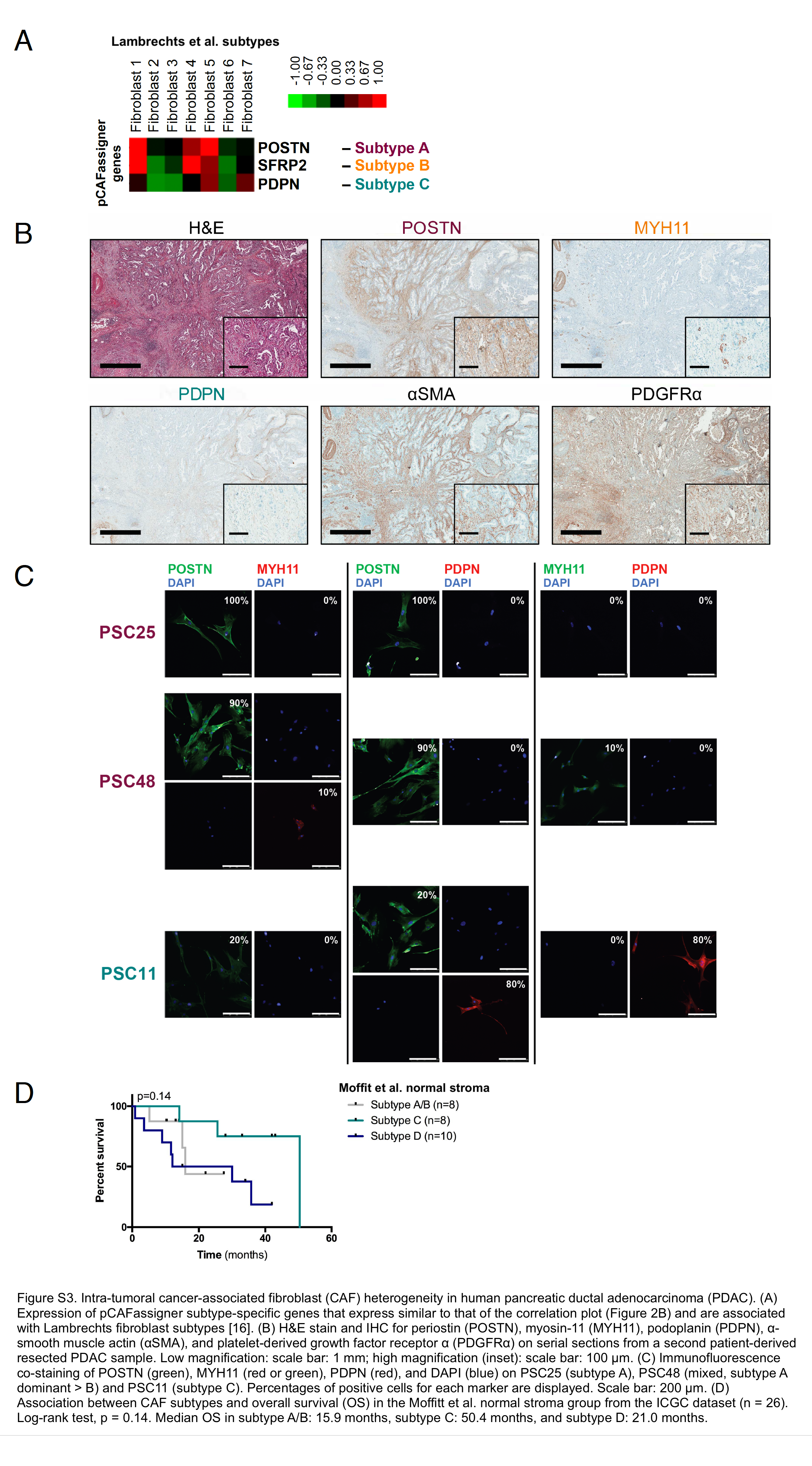

Supplement: Supplementary file 5 — Figure S3. Intra‐tumoural CAF heterogeneity in human PDAC. (A) Expression of pCAFassigner subtype‐specific genes that express similar to that of the correlation plot (Figure 2B) and are associated with Lambrechts fibroblast subtypes 16. (B) H&E stain and IHC for periostin (POSTN), myosin‐11 (MYH11), podoplanin (PDPN), αSMA and PDGFRα on serial sections from a second patient‐derived resected PDAC sample. Low magnification: scale bar: 1 mm; high magnification (inset): scale bar: 100 μm. (C) Immunofluorescence co‐staining of POSTN (green), MYH11 (red or green), PDPN (red) and DAPI (blue) on PSC25 (subtype A), PSC48 (mixed, subtype A dominant > B) and PSC11 (subtype C). Percentages of positive cells for each marker are displayed. Scale bar: 200 μm. (D) Association between CAF subtypes and OS in the Moffitt et al normal stroma group from the ICGC dataset (n = 26). Log‐rank test, p = 0.14. Median OS in subtype A/B: 15.9 months, subtype C: 50.4 months and subtype D: 21.0 months. [file PATH-248-51-s005.tif]

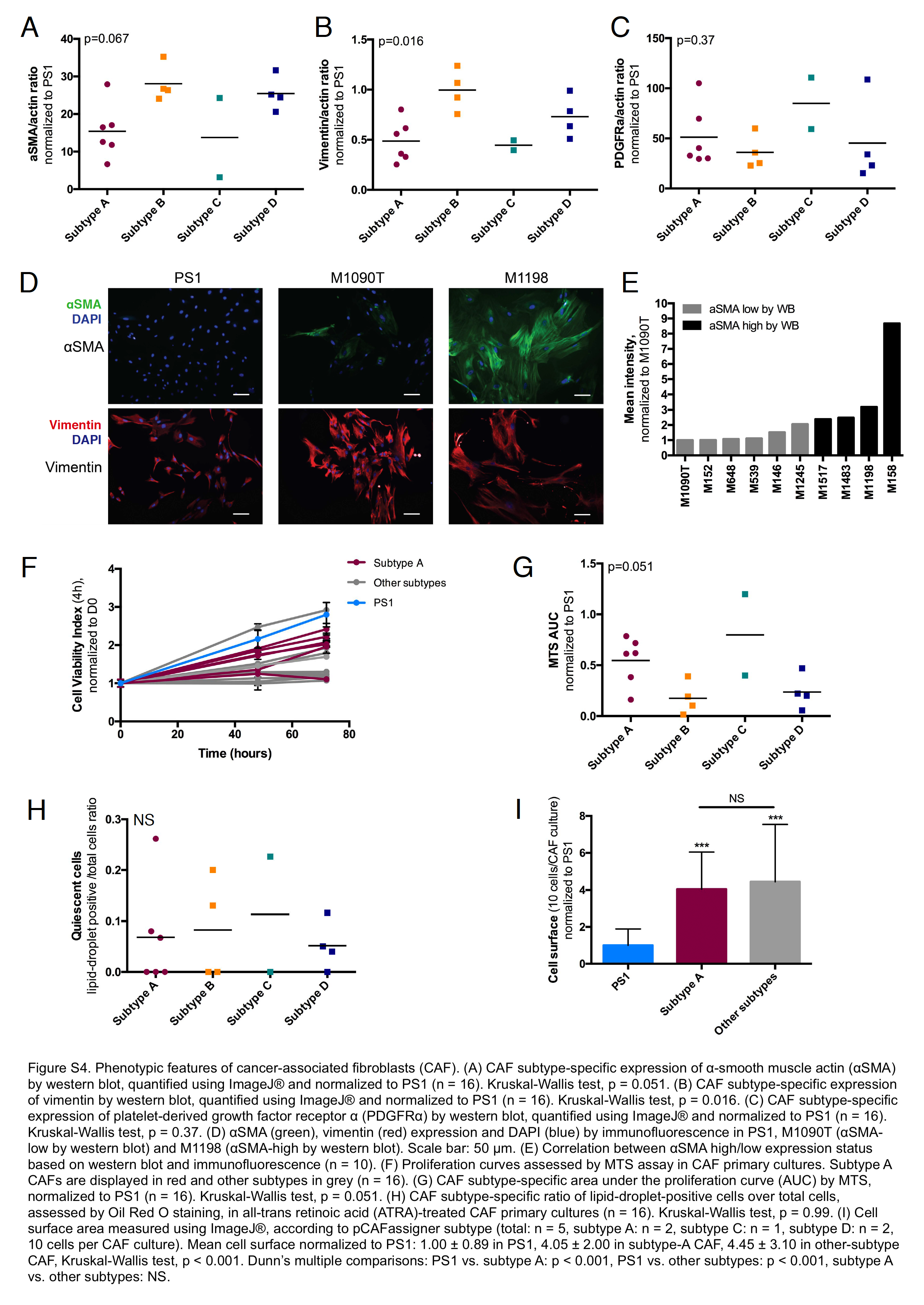

Supplement: Supplementary file 6 — Figure S4. Phenotypic features of CAF. (A) CAF subtype‐specific expression of αSMA by western blot, quantified using ImageJ and normalised to PS1 (n = 16). Kruskal–Wallis test, p = 0.051. (B) CAF subtype‐specific expression of vimentin by western blot, quantified using ImageJ and normalised to PS1 (n = 16). Kruskal–Wallis test, p = 0.016. (C) CAF subtype‐specific expression of platelet‐derived growth factor receptor α (PDGFRα) by western blot, quantified using ImageJ and normalised to PS1 (n = 16). Kruskal–Wallis test, p = 0.37. (D) αSMA (green), vimentin (red) expression and DAPI (blue) by immunofluorescence in PS1, M1090 T (αSMA‐low by western blot) and M1198 (αSMA‐high by western blot). Scale bar: 50 μm. (E) Correlation between αSMA high/low expression status based on western blot and immunofluorescence (n = 10). (F) Proliferation curves assessed by MTS assay in CAF primary cultures. Subtype A CAFs are displayed in red and other subtypes in grey (n = 16). (G) CAF subtype‐specific area under the proliferation curve (AUC) by MTS, normalised to PS1 (n = 16). Kruskal–Wallis test, p = 0.051. (H) CAF subtype‐specific ratio of lipid‐droplet‐positive cells over total cells, assessed by Oil Red O staining, in all‐trans retinoic acid (ATRA)‐treated CAF primary cultures (n = 16). Kruskal–Wallis test, p = 0.99. (I) Cell surface area measured using ImageJ, according to pCAFassigner subtype (total: n = 5, subtype A: n = 2, subtype C: n = 1, subtype D: n = 2, 10 cells per CAF culture). Mean cell surface normalised to PS1: 1.00 ± 0.89 in PS1, 4.05 ± 2.00 in subtype‐A CAF, 4.45 ± 3.10 in other‐subtype CAF, Kruskal–Wallis test, p < 0.001. Dunn's multiple comparisons: PS1 versus subtype A: p < 0.001, PS1 versus other subtypes: p < 0.001, subtype A versus other subtypes: NS. [file PATH-248-51-s006.tif]

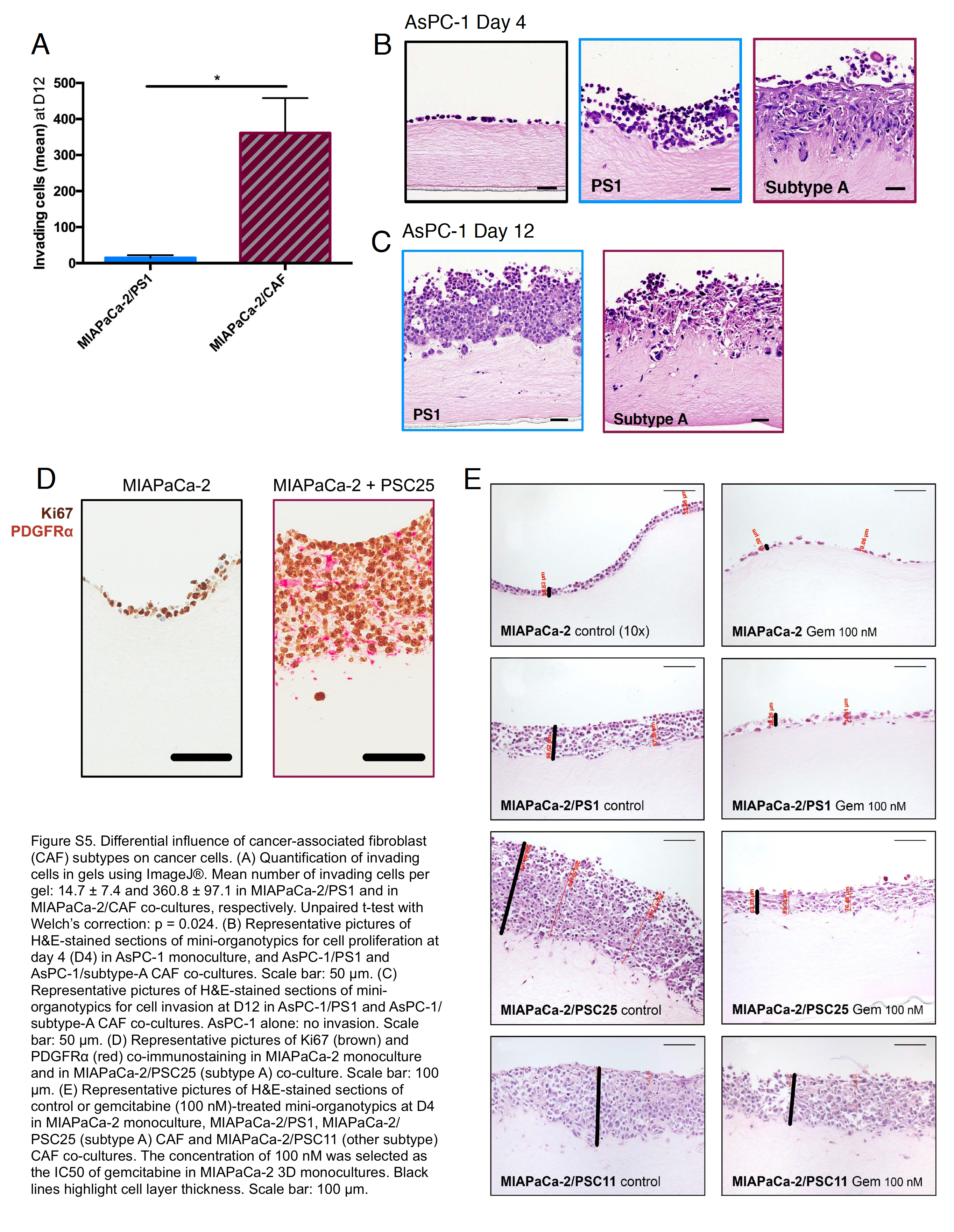

Supplement: Supplementary file 7 — Figure S5. Differential influence of CAF subtypes on cancer cells. (A) Quantification of invading cells in gels using ImageJ. Mean number of invading cells per gel: 14.7 ± 7.4 and 360.8 ± 97.1 in MIAPaCa‐2/PS1 and in MIAPaCa‐2/CAF co‐cultures, respectively. Unpaired t‐test with Welch's correction: p = 0.024. (B) Representative pictures of H&E‐stained sections of mini‐organotypics for cell proliferation at day 4 (D4) in AsPC‐1 monoculture, and AsPC‐1/PS1 and AsPC‐1/subtype‐A CAF co‐cultures. Scale bar: 50 μm. (C) Representative pictures of H&E‐stained sections of mini‐organotypics for cell invasion at D12 in AsPC‐1/PS1 and AsPC‐1/subtype‐A CAF co‐cultures. AsPC‐1 alone: no invasion. Scale bar: 50 μm. (D) Representative pictures of Ki67 (brown) and PDGFRα (red) co‐immunostaining in MIAPaCa‐2 monoculture and in MIAPaCa‐2/PSC25 (subtype A) co‐culture. Scale bar: 100 μm. (E) Representative pictures of H&E‐stained sections of control or gemcitabine (100 nm)‐treated mini‐organotypics at D4 in MIAPaCa‐2 monoculture, MIAPaCa‐2/PS1, MIAPaCa‐2/PSC25 (subtype A) CAF and MIAPaCa‐2/PSC11 (other subtype) CAF co‐cultures. The concentration of 100 nm was selected as the IC50 of gemcitabine in MIAPaCa‐2 3D monocultures. Black lines highlight cell layer thickness. Scale bar: 100 μm. [file PATH-248-51-s007.tif]

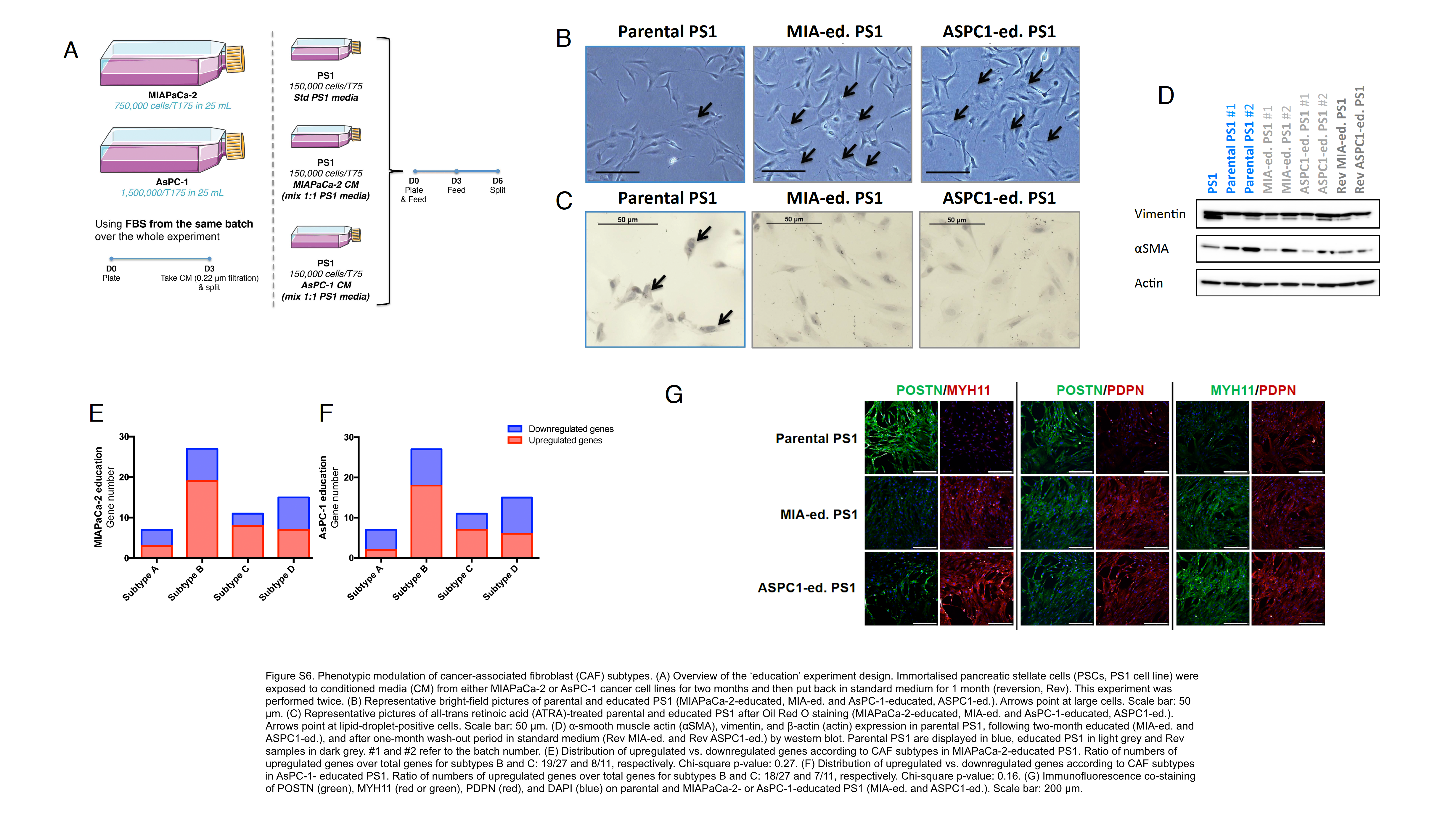

Supplement: Supplementary file 8 — Figure S6. Phenotypic modulation of CAF subtypes. (A) Overview of the ‘education’ experiment design. Immortalised pancreatic stellate cells (PSCs, PS1 cell line) were exposed to conditioned media (CM) from either MIAPaCa‐2 or AsPC‐1 cancer cell lines for 2 months and then put back in standard medium for 1 month (reversion, Rev). This experiment was performed twice. (B) Representative bright‐field pictures of parental and educated PS1 (MIAPaCa‐2‐educated, MIA‐ed. and AsPC‐1‐educated, ASPC1‐ed.). Arrows point at large cells. Scale bar: 50 μm. (C) Representative pictures of all‐trans retinoic acid (ATRA)‐treated parental and educated PS1 after Oil Red O staining (MIAPaCa‐2‐educated, MIA‐ed. and AsPC‐1‐educated, ASPC1‐ed.). Arrows point at lipid‐droplet‐positive cells. Scale bar: 50 μm. (D) αSMA, vimentin and β‐actin (actin) expression in parental PS1, following 2‐month educated (MIA‐ed. and ASPC1‐ed.), and after 1‐month wash‐out period in standard medium (Rev MIA‐ed. and Rev ASPC1‐ed.) by western blot. Parental PS1 are displayed in blue, educated PS1 in light grey and Rev samples in dark grey. #1 and #2 refer to the batch number. (E) Distribution of up‐regulated versus down‐regulated genes according to CAF subtypes in MIAPaCa‐2‐educated PS1. Ratio of numbers of up‐regulated genes over total genes for subtypes B and C: 19/27 and 8/11, respectively. Chi‐square P value: 0.27. (F) Distribution of up‐regulated versus down‐regulated genes according to CAF subtypes in AsPC‐1‐ educated PS1. Ratio of numbers of up‐regulated genes over total genes for subtypes B and C: 18/27 and 7/11, respectively. Chi‐square P value: 0.16. (G) Immunofluorescence co‐staining of POSTN (green), MYH11 (red or green), PDPN (red) and DAPI (blue) on parental and MIAPaCa‐2‐ or AsPC‐1‐educated PS1 (MIA‐ed. and ASPC1‐ed.). Scale bar: 200 μm. [file PATH-248-51-s008.tif]
