# Supplementary material for: Inter‐ and intra‐tumoural heterogeneity in cancer‐associated fibroblasts of human pancreatic ductal adenocarcinoma
Source: J Pathol. 2019 Feb 22;248(1):51–65. doi: 10.1002/path.5224 (PMC6492001; doi:10.1002/path.5224)
Supplement: Supplementary file 9 — Table S1. Clinico‐pathological characteristics of the 16 tumours used for primary CAF culture isolation Table S2. KRAS mutation status Table S3. pCAFassigner subtype assignment in the ICGC dataset Table S4. Summary of ICGC sample distribution according to first and second CAF subtypes Table S5. Classification of the 50 evaluable samples (IHC cohort) based on POSTN, MYH11 and PDPN expression levels Table S6. Contingency table of up‐regulated or down‐regulated genes following education of MIAPaCa‐2 or AsPC‐1 cells Table S7. Gene expression pathway analyses in in vitro educated PS1 cells Table S8. Culture media and conditions for cell lines Table S9. Antibodies used for western blotting Table S10. Antibodies used for immunofluorescence Table S11. Antibodies used for immunohistochemistry [file PATH-248-51-s002.docx]

**Inter- and intra-tumoural heterogeneity in cancer-associated fibroblasts of human pancreatic ductal adenocarcinoma**

Neuzillet C *et al*. *J Pathol* 2018 (DOI: 10.1002/path.5224)

**Table S1.** Clinico-pathological characteristics of the 16 tumours used for primary CAF culture isolation

| **CAF primary culture** | **T** | **N** | **M** | **G** | **R** | **CAF subtype** |
| --- | --- | --- | --- | --- | --- | --- |
| **PSC05** | 3 | 1 | 0 | NA | 1 | D |
| **PSC11** | 3 | 1 | 0 | 3 | 1 | C |
| **PSC25** | NA | NA | NA | NA | NA | A |
| **PSC46** | NA | 1 | 0 | 3 | NA | D |
| **PSC48** | 3 | 1 | 0 | NA | NA | A |
| **M146** | 3 | 0 | 1 | 3 | 0 | A |
| **M152** | 3 | 1 | 0 | 2 | 1 | B |
| **M158** | 3 | 0 | 1 | 4 | 0 | B |
| **M539** | 3 | 0 | 0 | 3 | 1 | C |
| **M648** | 4 | 1 | 0 | 2 | NA | D |
| **M1090T** | 3 | 1 | 0 | 2 | 0 | A |
| **M1092** | 3 | 1 | 1 | 3 | NA | B |
| **M1198** | 3 | 1 | 0 | 4 | 1 | B |
| **M1245** | 3 | 1 | 0 | 3 | 0 | A |
| **M1483** | 3 | 1 | 0 | 2 | 1 | A |
| **M1517** | 3 | 1 | 0 | 2 | NA | D |

T: tumour TNM stage; N: lymph node TNM stage; M: metastasis TNM stage; G: grade; R: resection margin (0: negative, 1: positive); NA: not available

**Table S2.** *KRAS* mutation status

| **Cell line or CAF primary culture** | **Codon 12** | **Codon 13** | **Codon 61** |
| --- | --- | --- | --- |
| **MIAPaCa-2** | Homozygous Mut c.34G>T (p.G12C) | WT | WT |
| **AsPC-1** | Homozygous Mut c.35G>A (p.G12D) | WT | WT |
| **MRC5** | WT | WT | WT |
| **PS1** | WT | WT | WT |
| **PSC05** | WT | WT | WT |
| **PSC11** | WT | WT | WT |
| **PSC25** | WT | WT | WT |
| **PSC46** | WT | WT | WT |
| **PSC48** | WT | WT | WT |
| **M146** | WT | WT | WT |
| **M152** | WT | WT | WT |
| **M158** | WT | WT | WT |
| **M539** | WT | WT | WT |
| **M648** | WT | WT | WT |
| **M1090T** | WT | WT | WT |
| **M1092** | WT | WT | WT |
| **M1198** | WT | WT | WT |
| **M1245** | WT | WT | WT |
| **M1483** | WT | WT | WT |
| **M1517** | WT | WT | WT |

Mut: mutation; WT: wild-type

**Table S3.** pCAFassigner subtype assignment in the ICGC dataset. Information from the publicly available International Cancer Genome Consortium dataset [12] with clinico-pathological details (n = 70 PDAC). NA: Not assessable.

| **Sample ID** | **First subtype** | **Second subtype** | **Third subtype** | **Ethnicity** | **Sex** | **Age (y)** | **Country** | **Smoking** | **Tumour grade** | **Tumour location** | **AJCC stage** | **Alive (0) or dead (1)** | **Follow-up (months)** |
| --- | --- | --- | --- | --- | --- | --- | --- | --- | --- | --- | --- | --- | --- |
| ICGC_0535 | A | NA | NA | White / Caucasian | F | 40 | Australia | NA | 3 | Head | IIB | 0 | 9.4 |
| ICGC_0031 | A | NA | NA | White / Caucasian | F | 61 | Australia | Stopped Smoking | 2 | Head | IIB | 1 | 35.9 |
| ICGC_0067 | A | NA | NA | White / Caucasian | M | 64 | Australia | Never Smoked | 3 | Head | IIB | 1 | 16.5 |
| ICGC_0392 | A | NA | NA | Not documented | M | 50 | USA | NA | 3 | Head | IIB | 1 | 10.9 |
| ICGC_0300 | B | NA | NA | White / Caucasian | F | 72 | Australia | Never Smoked | 1 | Head | IIA | 0 | 22 |
| ICGC_0052 | C | NA | NA | White / Caucasian | M | 76 | Australia | Never Smoked | 2 | Head | IIA | 0 | 47 |
| ICGC_0301 | C | NA | NA | Asian | M | 68 | Australia | Never Smoked | 2 | Head | IIB | 0 | 18 |
| ICGC_0188 | D | NA | NA | Asian | M | 72 | Australia | Stopped Smoking | 3 | Body | IIB | 1 | 7.8 |
| ICGC_0326 | D | NA | NA | White / Caucasian | F | 86 | Australia | Never Smoked | 2 | Tail | IIA | 1 | 9 |
| ICGC_0354 | D | NA | NA | White / Caucasian | M | 49 | Australia | Still Smoking | 3 | Head | IV | 1 | 3.6 |
| ICGC_0518 | D | NA | NA | White / Caucasian | M | 58 | UK | NA | 2 | Head | IIB | 1 | 0.9 |
| ICGC_0227 | A | D | NA | White / Caucasian | F | 49 | Australia | Still Smoking | 3 | Head | IIB | 1 | 9.5 |
| ICGC_0503 | B | D | NA | White / Caucasian | F | 74 | UK | NA | 2 | Head | IIB | 0 | 27.5 |
| ICGC_0212 | B | D | NA | Asian | M | 57 | USA | Occasional | 2 | Head | IIB | 1 | 19.8 |
| ICGC_0486 | B | D | NA | White / Caucasian | F | 77 | Australia | Never Smoked | 3 | Head | IIB | 1 | 5 |
| ICGC_0522 | B | D | NA | White / Caucasian | F | 56 | UK | NA | 3 | Head | IIB | 1 | 15 |
| ICGC_0059 | C | D | NA | White / Caucasian | M | 68 | Australia | Stopped Smoking | 2 | Head | IIB | 0 | 42 |
| ICGC_0169 | C | D | NA | White / Caucasian | F | 77 | USA | Stopped Smoking | 3 | Head | IIB | 0 | 28 |
| ICGC_0185 | C | D | NA | Black / African | F | 61 | Australia | Still Smoking | 2 | Head | IIB | 0 | 33 |
| ICGC_0146 | C | D | NA | White / Caucasian | M | 52 | Australia | Never Smoked | 3 | Head | IB | 0 | 35 |
| ICGC_0507 | C | D | NA | White / Caucasian | M | 77 | UK | NA | 2 | Head | IIB | 1 | 50.4 |
| ICGC_0296 | A | C | NA | White / Caucasian | M | 75 | Australia | Stopped Smoking | 3 | Head | IIA | 0 | 24 |
| ICGC_0304 | A | C | NA | White / Caucasian | F | 67 | Australia | Stopped Smoking | 2 | Head | IIB | 0 | 24 |
| ICGC_0115 | A | C | NA | White / Caucasian | F | 70 | Australia | Stopped Smoking | 2 | Head | IIB | 1 | 26.5 |
| ICGC_0199 | A | C | NA | White / Caucasian | M | 77 | Australia | Never Smoked | 3 | Head | IIB | 1 | 17.7 |
| ICGC_0206 | A | C | NA | White / Caucasian | F | 90 | USA | Never Smoked | 3 | Tail | IIB | 1 | 3.6 |
| ICGC_0415 | B | C | NA | Asian, White | F | 55 | Australia | Stopped Smoking | 2 | Head | IIB | 0 | 10.3 |
| ICGC_0075 | B | C | NA | White / Caucasian | F | 76 | Australia | Never Smoked | 2 | Head | IIB | 1 | 15.9 |
| ICGC_0124 | B | C | NA | White / Caucasian | M | 79 | Australia | Stopped Smoking | 2 | Head | IIB | 1 | 5.2 |
| ICGC_0153 | B | C | NA | White / Caucasian | F | 83 | Australia | Stopped Smoking | 2 | Head | IIB | 1 | 13.7 |
| ICGC_0521 | B | C | NA | White / Caucasian | F | 51 | UK | NA | 2 | Tail | IV | 1 | 14.1 |
| ICGC_0048 | D | C | NA | White / Caucasian | F | 65 | Australia | Never Smoked | 2 | Head | IIA | 0 | 42 |
| ICGC_0087 | D | C | NA | White / Caucasian | M | 70 | Australia | Stopped Smoking | 2 | Head | IIB | 1 | 12 |
| ICGC_0103 | D | C | NA | White / Caucasian | F | 78 | Australia | Stopped Smoking | 2 | Tail | IIB | 1 | 35.8 |
| ICGC_0109 | D | C | NA | White / Caucasian | M | 70 | Australia | Still Smoking | 3 | Head | IIA | 1 | 30 |
| ICGC_0201 | D | C | NA | White / Caucasian | F | 72 | Australia | Stopped Smoking | 2 | Head | IIB | 1 | 23.3 |
| ICGC_0207 | A | B | NA | White / Caucasian | M | 80 | USA | Stopped Smoking | 2 | Head | IB | 1 | 6.8 |
| ICGC_0536 | A | B | NA | White / Caucasian | M | 58 | Australia | Still Smoking | 3 | Head | IIB | 1 | 5.7 |
| ICGC_0099 | A | B | NA | White / Caucasian | F | 80 | Australia | Never Smoked | 3 | Head | IIA | 1 | 0.3 |
| ICGC_0214 | C | B | NA | White / Caucasian | F | 82 | Australia | Never Smoked | 3 | Head | IIB | 1 | 13.2 |
| ICGC_0108 | C | B | NA | Not documented | M | 72 | Italy | NA | 3 | Head | IIB | 1 | 14.1 |
| ICGC_0417 | D | B | NA | Asian | M | 65 | Australia | Never Smoked | 2 | Head | IIA | 0 | 15 |
| ICGC_0139 | D | B | NA | White / Caucasian | M | 70 | Australia | Never Smoked | 2 | Head | IIB | 0 | 34 |
| ICGC_0205 | D | B | NA | White / Caucasian | M | 59 | USA | Stopped Smoking | 2 | Tail | IB | 1 | 9.4 |
| ICGC_0230 | D | B | NA | White / Caucasian | M | 37 | Australia | Still Smoking | 2 | Head | IIB | 1 | 8.4 |
| ICGC_0315 | D | B | NA | Asian | F | 58 | Australia | Never Smoked | NA | Head | III | 1 | 7.3 |
| ICGC_0526 | D | B | NA | White / Caucasian | M | 60 | UK | NA | 3 | Head | IIA | 1 | 0 |
| ICGC_0295 | B | A | NA | White / Caucasian | M | 60 | Australia | Stopped Smoking | 2 | Head | IIB | 0 | 21 |
| ICGC_0020 | B | A | NA | White / Caucasian | M | 57 | Australia | Stopped Smoking | 2 | Head | IIB | 1 | 42 |
| ICGC_0141 | B | A | NA | White / Caucasian | F | 75 | Australia | Never Smoked | 2 | Body | IIA | 1 | 12.9 |
| ICGC_0215 | B | A | NA | White / Caucasian | F | 79 | Australia | Stopped Smoking | 2 | Tail | IIA | 1 | 23.7 |
| ICGC_0135 | C | A | NA | Asian | F | 62 | Australia | Never Smoked | 2 | Head | IIA | 0 | 30 |
| ICGC_0144 | C | A | NA | White / Caucasian | M | 64 | Australia | Stopped Smoking | 2 | Head | IIA | 0 | 28 |
| ICGC_0321 | C | A | NA | White / Caucasian | M | 64 | USA | Still Smoking | 3 | Head | IB | 0 | 38 |
| ICGC_0025 | D | A | NA | White / Caucasian | M | 69 | Australia | Never Smoked | 3 | Head | IIB | 1 | 11.6 |
| ICGC_0066 | D | A | NA | White / Caucasian | M | 58 | Australia | Never Smoked | 2 | Head | IIB | 1 | 10.4 |
| ICGC_0412 | B | C | D | White / Caucasian | F | 77 | Australia | Never Smoked | 2 | Head | IIB | 0 | 13 |
| ICGC_0114 | A | B | D | White / Caucasian | F | 65 | USA | Stopped Smoking | 2 | Head | IIB | 1 | 16.6 |
| ICGC_0420 | C | A | D | White / Caucasian | M | 74 | Australia | Still Smoking | 3 | Head | IIB | 1 | 6.5 |
| ICGC_0021 | B | A | C | White / Caucasian | M | 60 | Australia | Stopped Smoking | 3 | Head | IIB | 1 | 23.8 |
| ICGC_0033 | C | D | B | White / Caucasian | M | 51 | Australia | Stopped Smoking | 3 | Head | IIB | 1 | 25.6 |
| ICGC_0365 | A | C | B | White / Caucasian | M | 50 | Australia | Never Smoked | 2 | Head | IIB | 0 | 11 |
| ICGC_0406 | A | C | B | White / Caucasian | F | 46 | Australia | Stopped Smoking | 2 | Head | IIA | 0 | 14 |
| ICGC_0007 | D | C | B | White / Caucasian | F | 74 | Australia | Never Smoked | 2 | Head | IIB | 1 | 1.9 |
| ICGC_0006 | C | A | B | White / Caucasian | F | 49 | Australia | Still Smoking | 3 | Body | IA | 0 | 43 |
| ICGC_0312 | C | A | B | White / Caucasian | M | 56 | Australia | Still Smoking | 2 | Tail | IIB | 1 | 14.1 |
| ICGC_0053 | C | D | A | White / Caucasian | F | 73 | Australia | Stopped Smoking | 2 | Head | IV | 1 | 20.3 |
| ICGC_0192 | C | D | A | White / Caucasian | M | 75 | Australia | Never Smoked | 2 | Tail | IIA | 1 | 3.3 |
| ICGC_0223 | C | B | A | White / Caucasian | F | 83 | Australia | NA | 2 | Head | IIB | 1 | 7.3 |
| ICGC_0026 | D | B | A | White / Caucasian | F | 69 | Australia | Stopped Smoking | 3 | Head | IIB | 1 | 8.7 |

**Table S4.** Summary of ICGC sample distribution according to first and second cancer-associated-fibroblast (CAF) subtypes. Total sample number: n = 70.

|  | **2^nd^ CAF subtype** | | | | | | |  |
| --- | --- | --- | --- | --- | --- | --- | --- | --- |
|  |  | **A** | **B** | **C** | | **D** | Total |  |
| **1^st^ CAF subtype** | **A** | 4 | 4 | 7 | | 1 | 16 |  |
|  | **B** | 5 | 1 | 6 | | 4 | 16 |  |
|  | **C** | 6 | 3 | 2 | | 8 | 19 |  |
|  | **D** | 2 | 7 | 6 | | 4 | 19 |  |
|  | Total | 17 | 15 | 21 | | 17 |  |  |
|  |  |  |  |  | |  |  |  |
|  |  | **Legend:** number of samples | | | | | | |
|  |  |  | ≤ 1 | |  |  |  |  |
|  |  |  | 2 | |  |  |  |  |
|  |  |  | 3 | |  |  |  |  |
|  |  |  | 4 | |  |  |  |  |
|  |  |  | 5 | |  |  |  |  |
|  |  |  | 6 | |  |  |  |  |
|  |  |  | ≥ 7 | |  |  |  |  |

**Table S5.** Classification of the 50 evaluable samples (IHC cohort) based on POSTN, MYH11 and PDPN expression levels. High periostin (POSTN) expression was defined as strong staining in >50% of stromal surface. High myosin-11 (MYH11) and podoplanin (PDPN) expressions were defined as the presence of strong stromal staining. POSTN-only tumours are highlighted in red, MYH11-only in orange, PDPN-only in green, mixed POSTN/MYH11 in yellow, mixed POSTN/PDPN in grey, and triple-negative samples in blue. The 10 double-positive MYH11/PDPN and triple-positive (unframed) samples were next reclassified into MYH11 or PDPN groups for survival analysis based on the most abundant CAF sub-population.

|  | **MYH11^high^/PDPN^low^** | **MYH11^low^/PDPN^high^** | **MYH11^high^/PDPN^high^** | **MYH11^low^/PDPN^low^** | Total |
| --- | --- | --- | --- | --- | --- |
| **POSTN^low^** | 4 | 7 | 7 | 5 | 23 |
| **POSTN^high^** | 1 | 10 | 4 | 12 | 27 |
| Total | 5 | 17 | 11 | 17 |  |

**Table S6.** Contingency table of up-regulated or down-regulated genes following education of MIAPaCa-2 or AsPC-1 cells. Genes up-regulated (UP) or down-regulated (DOWN) following MIAPaCa-2- (MIA-ed.) or AsPC-1-education (ASPC1-ed.) versus parental PS1 are enumerated. Genes specifically upregulated in MIAPaCa-2-educated PS1 (n=6) are listed and highlighted with red background, and genes specifically upregulated in AsPC-1-educated PS1 (n=2) are listed and highlighted with green background. Fisher’s exact test, p < 0.0001.

|  | **ASPC1-ed. UP** | **ASPC1-ed. DOWN** | |
| --- | --- | --- | --- |
| **MIA-ed. UP** | **N=31**  **[Table S7]** | N=6 | |
|  |  | *- TNC - CRISPLD2*  *- ANGPTL4* | *- IL13RA2*  *- EREG - ADM2* |
| **MIA-ed. DOWN** | N=2  *- TF*  *- NRXN3* | **N=21**  **[Table S7]** | |

**Table S7.** Gene expression pathway analyses in *in vitro* educated PS1 cells. Gene expression pathway analyses using the Broad Institute mSigDB database [54] are shown. Geneset lists for genes that were upregulated (n = 31) or downregulated (n = 21) in both MIAPaCa-2- and AsPC-1-educated PS1 *vs.* parental PS1. Top 20 pathways with *p* < 0.05 are displayed. Extra-cellular matrix (ECM) production/degradation pathways are shown in bold type, and immune-related (subtype-C like) pathways are in green.

| **UP-regulated genes** (n = 31) | **DOWN-regulated genes** (n = 21) |
| --- | --- |
| BIOCARTA LAIR (Local Acute Inflammatory Response) Pathway  PID AP1 Pathway  PID Regulatory Glucocorticoid Receptor Pathway  KEGG Cell Adhesion Molecules  PID Fra Pathway  KEGG Pathways in Cancer  **NABA Matrisome**  REACTOME Cell Junction Organization  BIOCARTA Lymphocyte Pathway  BIOCARTA Granulocyte Pathway  BIOCARTA Stem Pathway  BIOCARTA IL17 Pathway  REACTOME Cell-Cell Communication  BIOCARTA Cytokine Pathway  **NABA Matrisome Associated**  BIOCARTA Inflammation Pathway  PID Integrin2 Pathway  **REACTOME Degradation of the ECM**  PID AMB2 Neutrophil Pathway  KEGG Bladder Cancer | **NABA Matrisome**  **NABA Core Matrisome**  **NAMA ECM Glycoproteins** |

**Table S8.** Culture media and conditions for cell lines

| **Cell line** | **Culture media** | **Incubator conditions** |
| --- | --- | --- |
| **MIAPaCa-2** | DMEM + 10% FBS | 37 °C / 5% CO_2_ |
| **AsPC-1** | RPMI + 10% FBS | 37 °C / 5% CO_2_ |
| **PS1** | PS1 medium = mixed DMEM (50%) / F12 (50%) medium + 10% FBS + puromycin (1 μg/ml) | 37 °C / 5% CO_2_ |
| **MRC5** | αMEM + 10% FBS | 37 °C / 5% CO_2_ |
| **Primary CAFs** | DMEM + 20% FBS until passage 2-3, then DMEM + 10% FBS for characterisation and up to 10–15 passages | 37 °C / 5% CO_2_ |

CAF: cancer-associated fibroblasts; FBS: foetal bovine serum

**Table S9.** Antibodies used for western blotting

| **Antibody to** | **Species raised in** | **Supplier (catalogue no.)** | **Dilution** |
| --- | --- | --- | --- |
| **αSMA** | Rabbit | Abcam (ab5694) | 1:200 |
| **vimentin** | Rabbit | Cell Signaling (5741) | 1:1,000 |
| **PDGFRα** | Rabbit | Cell Signaling (5241) | 1:750 |
| **β-actin** | Mouse | Sigma-Aldrich (A544) | 1:500 |
| **anti-mouse:HRP** | Horse | Cell Signaling (7076) | 1:1,000 |
| **anti-rabbit:HRP** | Goat | Cell Signaling (7074) | 1:1,000 |

αSMA: α-smooth muscle actin, HRP: horseradish peroxidase, PDGFRα: platelet-derived growth factor receptor α.

**Table S10.** Antibodies used for immunofluorescence

| **Antibody** | **Species raised in** | **Supplier (catalogue no.)** | **Dilution** |
| --- | --- | --- | --- |
| **αSMA** | Mouse | Sigma (F3777) | 1:500 |
| **GFAP** | Mouse | Sigma (G3893) | 1:500 |
| **desmin** | Mouse | Sigma (D1033) | 1:100 |
| **vimentin** | Rabbit | Atlas (HPA001762) | 1:50 |
| **periostin (POSTN)** | Rat | R&D systems (MAB3548) | 1:10 |
| **myosin 11 (MYH11)** | Rabbit | Sigma (HPA015310) | 1:60 |
| **podoplanin (PDPN)** | Mouse | Clinisciences (BTMC-A254-3) | 1:10 |
| **IgG1** | Mouse | Dako (X0931) | 1:10 |
| **IgG** | Rabbit | Abcam (ab172730) | 1:100 |
| **anti-mouse A488** | Goat | Invitrogen (A11017) | 1:500 |
| **anti-rabbit A546** | Goat | Invitrogen (A11035) | 1:500 |
| **anti-rat A488** | Donkey | Life Technologies | 1:200 |
| **anti-mouse A488** | Goat | Life Technologies | 1:200 |
| **anti-mouse A555** | Goat | Life Technologies | 1:200 |
| **anti-rabbit A555** | Goat | Life Technologies | 1:200 |

αSMA: α-smooth muscle actin, GFAP: glial fibrillary acidic protein, IgG: immunoglobulin G.

**Table S11.** Antibodies used for immunohistochemistry

| **Primary antibody to** | **Species raised in** | **Supplier (catalogue no.)** | **Dilution** | **Detection** |
| --- | --- | --- | --- | --- |
| **periostin (POSTN)** | Rabbit | Abcam (ab219057) | 1:500 | iVIEW DAB Detection Kit, Ventana, Roche |
| **myosin 11 (MYH11)** | Rabbit | Sigma (HPA015310) | 1:200 |  |
| **podoplanin (PDPN)** | Rabbit | Sigma (HPA007534) | 1:250 |  |
| **αSMA** | Mouse | Dako (M0851) | 1:600 |  |
| **PDGFRα** | Rabbit | Cell Signaling (3174) | 1:100 |  |
| **Ki67** | Mouse | Dako (M7240) | 1:200 |  |
